# Mechanisms of action of ribosomally synthesized and posttranslationally modified peptides (RiPPs)

**DOI:** 10.1093/jimb/kuab005

**Published:** 2021-01-27

**Authors:** Li Cao, Truc Do, A James Link

**Affiliations:** Department of Chemical and Biological Engineering, Princeton University, Princeton, NJ 08544, USA; Department of Chemical and Biological Engineering, Princeton University, Princeton, NJ 08544, USA; Department of Chemical and Biological Engineering, Princeton University, Princeton, NJ 08544, USA; Department of Chemistry, Princeton University, Princeton, NJ 08544, USA; Department of Molecular Biology, Princeton University, Princeton, NJ 08544, USA

**Keywords:** RiPPs, Natural products, Mechanism of action

## Abstract

Natural products remain a critical source of medicines and drug leads. One of the most rapidly growing superclasses of natural products is RiPPs: ribosomally synthesized and posttranslationally modified peptides. RiPPs have rich and diverse bioactivities. This review highlights examples of the molecular mechanisms of action that underly those bioactivities. Particular emphasis is placed on RiPP/target interactions for which there is structural information. This detailed mechanism of action work is critical toward the development of RiPPs as therapeutics and can also be used to prioritize hits in RiPP genome mining studies.

## Introduction

**Fig. 1.**
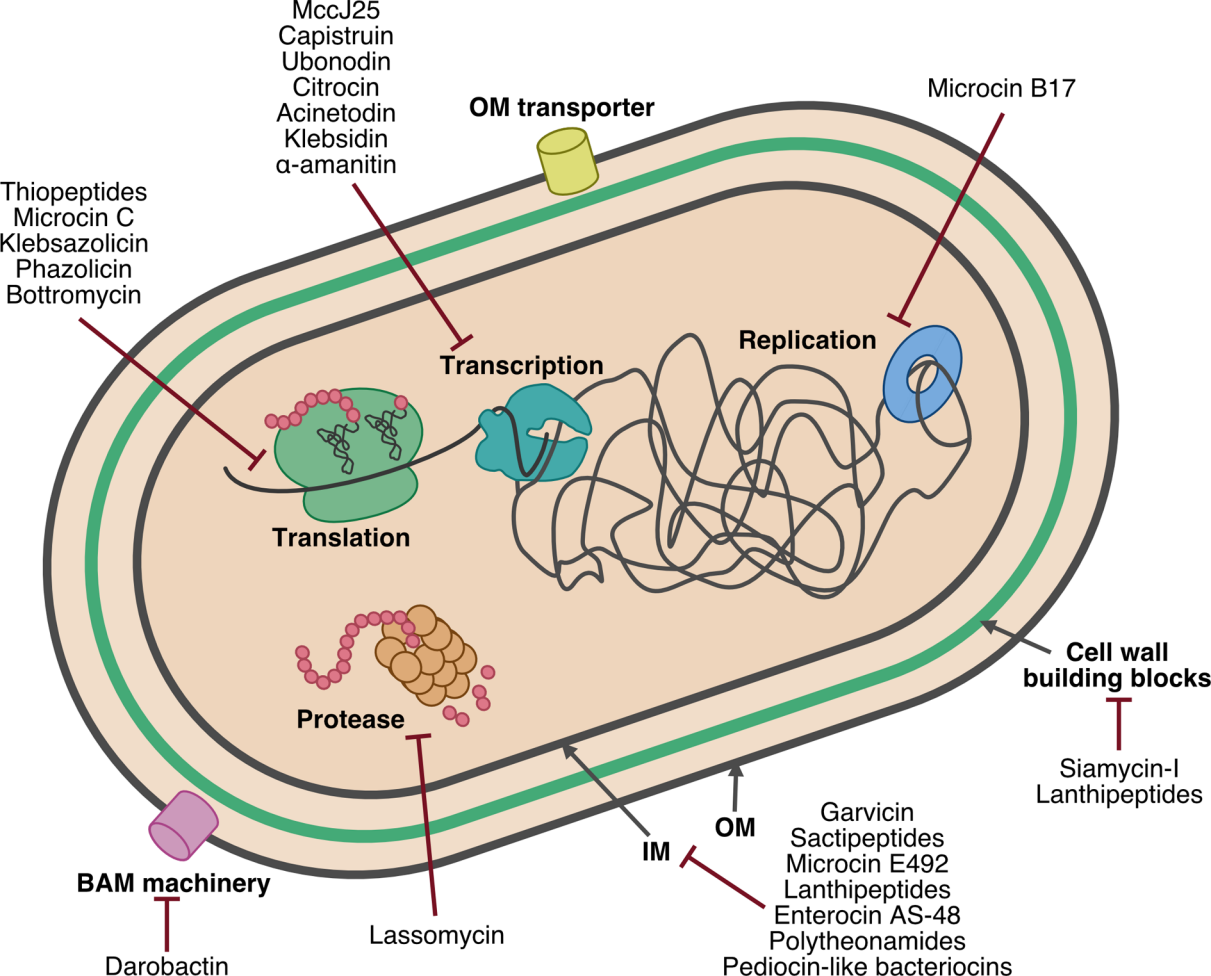
The varied targets of bioactive RiPPs. Members of the RiPPs superfamily exert antibiotic and/or cytotoxic activity via inhibition of nearly all major cellular processes. Many RiPPs are membrane-active, compromising the permeability barrier of the cytoplasmic membrane in both Gram-negative and Gram-positive bacteria (only a Gram-negative cell is shown for simplicity's sake). Other RiPPs active at the cell envelope disrupt outer membrane biogenesis in Gram-negative bacteria and peptidoglycan (cell wall) biosynthesis. RiPPs also engage cytoplasmic targets disrupting the central processes of DNA replication, transcription, and translation. OM: outer membrane, IM: inner membrane.

Much of our current pharmaceutical arsenal owes its existence to natural products, molecules produced by bacteria, fungi, and plants (Li & Vederas, 2009). In addition to their utility in medicine, natural products have also captured the interest of scientists from a variety of disciplines. For decades, synthetic chemists have focused on recapitulating the architectural and stereochemical complexity of natural products in the laboratory while enzymologists have studied the intricate steps of natural product biosynthesis. More recently, with the exponential rise of sequencing data in the 21st century, the field of bioinformatics has played an increasingly prominent role in natural products discovery. Great strides have been made in predicting the structure of a natural product based on the gene cluster that encodes its biosynthesis (Blin, Pascal Andreu, et al., 2019; Blin, Shaw, et al., 2019). One superfamily of natural products that has particularly benefited from “genome mining” approaches is the RiPPs: ribosomally synthesized and posttranslationally modified peptides (Arnison et al., 2013). The biosynthesis of RiPPs starts with a gene-encoded precursor peptide, and the gene encoding this precursor is often colocalized with genes encoding the enzymes that mature it into the final natural product. Many powerful software packages have been developed to identify RiPP biosynthetic gene clusters (BGCs) from genomic sequence data (Russell & Truman, 2020). This has led to the rapid discovery of novel RiPP chemistry and biosynthesis, subjects that have been extensively and authoritatively covered in a recent review paper (Montalbán-López et al., 2021). Thus, we provide only basic information about RiPP structure and biosynthesis below. Li and Rebuffat have also recently reviewed the roles of RiPPs in physiology and ecology (Li & Rebuffat, 2020). Here, we review the molecular mechanisms of action of select RiPPs with a particular focus on RiPPs with either antimicrobial or cytotoxic activity. RiPPs exert these activities by targeting key cellular processes such as DNA replication, transcription, and translation (Travin et al., 2020). RiPPs also interact extensively with the cell envelope in both Gram-negative and Gram-positive bacteria (Fig. 1). Rather than organizing this review by RiPP families, we have organized it into sections based on the targets of the RiPPs. In this way, both commonalities and diversity in RiPP mechanisms of action can be appreciated.

## RiPPs Targeting the Cell Envelope

### Lanthipeptides that Attack the Bacterial Cell Envelope

**Fig. 2.**
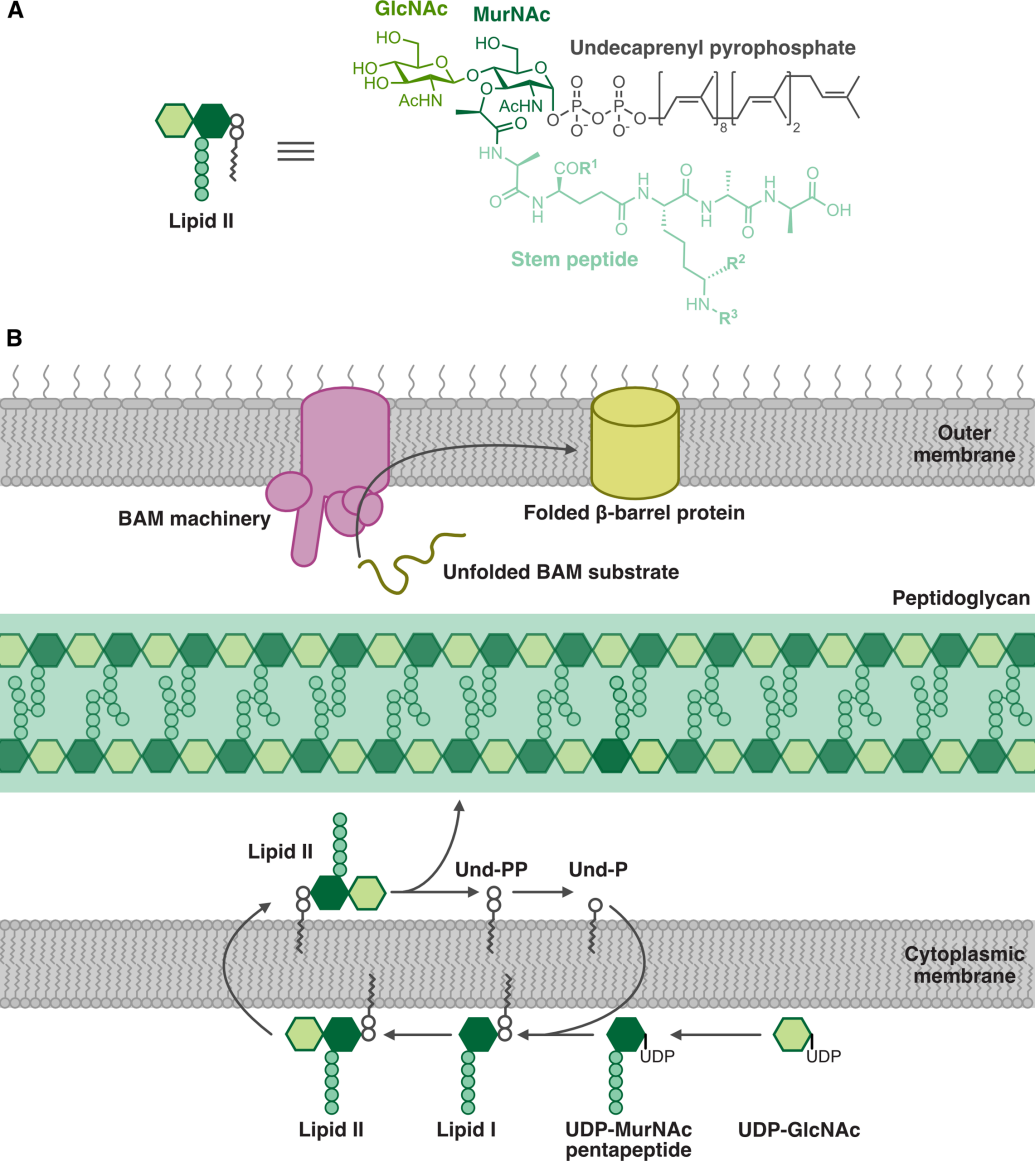
The bacterial cell envelope is a common target for RiPPs. (A) Cartoon and chemical structure of lipid II, the precursor to peptidoglycan. The stem peptides of lipid II from different bacteria vary at the positions indicated by R^1^, R^2^, and R^3^. GlcNAc: *N*-acetylglucosamine, MurNAc: *N*-acetylmuramic acid. (B) Schematic of cell envelope biosynthesis processes disrupted by RiPPs. Both lanthipeptides and the lasso peptide siamycin-I bind to lipid II, disrupting peptidoglycan biosynthesis. The recently discovered RiPP darobactin inhibits BamA, disrupting the assembly of outer membrane proteins in Gram-negative bacteria. BAM: β-barrel assembly machinery.

A major target of RiPPs is the bacterial cell envelope, an intricate, multilayered structure that surrounds and protects bacterial cells from their environment (Silhavy et al., 2010). The integrity of the cell envelope is central to bacterial viability and a breach in this structure oftentimes leads to cell death. As such, drugs that cripple cell envelope biogenesis are highly sought-after because the pathway is essential and unique to bacteria and many cell envelope targets are accessible from outside the cell. Broadly, there are two types of bacterial cell envelope, those belonging to Gram-negative versus Gram-positive organisms. The Gram-negative cell envelope comprises an inner and outer membrane with a thin layer of peptidoglycan sandwiched between the membranes (Fig. 2). Gram-positive bacteria lack an outer membrane and instead are encased in a thick layer of peptidoglycan that sits above the cytoplasmic membrane. Many cell envelope-targeting RiPPs are thought to be pore-forming peptides that penetrate the cytoplasmic membrane. An important step in engagement of these RiPPs with the cell membrane is oftentimes recognition of key intermediates in the biosynthesis of the peptidoglycan cell wall.

Peptidoglycan is a crosslinked glycopolymer of repeating disaccharide-peptide units that is assembled from a basic building block called lipid II (Fig. 2A) (Vollmer et al., 2008). Despite the diversity of bacteria, the lipid II molecules that make up their cell wall are chemically conserved. Lipid II contains a disaccharide of *N*-acetylmuramic acid (MurNAc) and *N*-acetylglucosamine (GlcNAc). An undecaprenyl pyrophosphate lipid tail is linked to the anomeric carbon of MurNAc and a short peptide side chain is covalently attached to the MurNAc C3 carbon. The identity of the amino acids that comprise the peptide side chain can vary in different bacteria, but for the most part is similar across many species. Lipid II is synthesized inside the cell and the fully assembled molecule is then translocated across the cytoplasmic membrane to the outer leaflet, where peptidoglycan glycosyltransferase enzymes polymerize the units into glycan strands (Fig. 2B) (Bouhss et al., 2008; Egan et al., 2020; Typas et al., 2012; van Heijenoort, 2007). To reinforce the cell wall matrix, enzymes with peptidoglycan transpeptidase function form crosslinks between the peptide side chains of the strands (Sauvage et al., 2008). The conserved chemistry and synthesis of cell wall intermediates make them highly effective antibiotic targets.

In 1928, around the time that penicillin was discovered, one of the first antimicrobial RiPPs was also found (Rogers, 1928), though the fact that it was ribosomally synthesized was not confirmed until much later. This RiPP, called nisin, is a lanthipeptide from *Lactococcus lactis* that is present in fermented milk. Nisin has been used as a food preservative with broad-spectrum antimicrobial activity for decades, but only recently have we begun to understand its mechanism of action (McAuliffe et al., 2001). Early studies reported that nisin caused membrane leakage similar to a detergent and inhibited cell wall synthesis (Breukink et al., 1999; Linnett & Strominger, 1973; Van Heusden et al., 2002; Wiedemann et al., 2004). Advances in structural biology tools and access to defined cell wall substrates now have enabled studies clarifying that nisin docks on lipid II and thereafter permeabilizes the cytoplasmic membrane, leading to changes in the membrane potential and cell death (Breukink et al., 2003; Hsu et al., 2004; ‘t Hart et al., 2016).

**Fig. 3.**
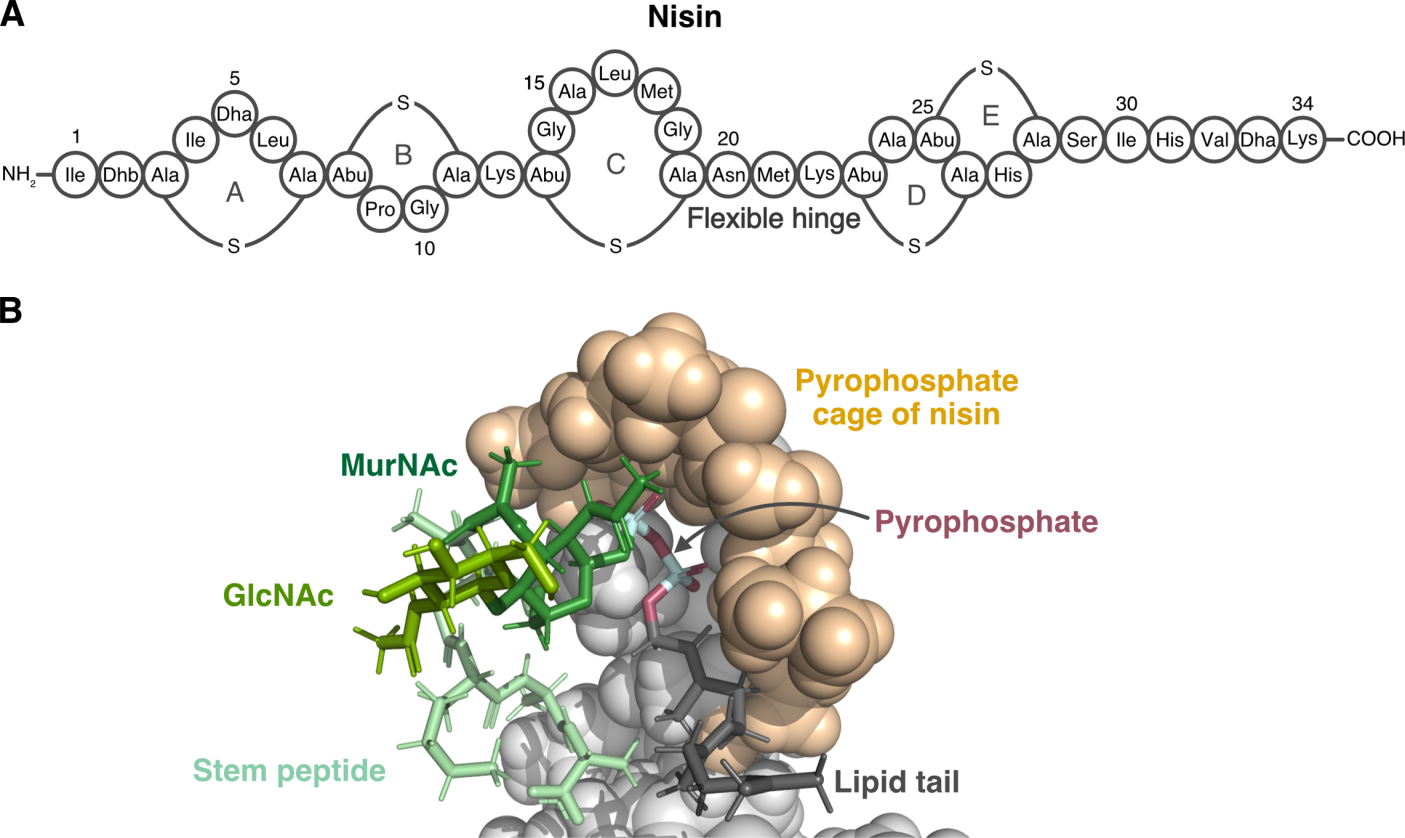
Structure and mechanism of action of the lanthipeptide nisin. (A) Representation of nisin structure. Dhb: dehydrobutyrine, Dha: dehydroalanine, Abu: aminobutyric acid. The thioether linkage between Ala and Ala is referred to as a lanthionine moiety while the linkage between Abu and Ala is a methyllanthionine linkage. (B) NMR structure (PDB file 1WCO) of nisin bound to a lipid II analog. Nisin is shown as a space-filling model and the lipid II analog is shown as sticks. The N-terminal (methyl)lanthionine rings (space-filling tan) of nisin envelop the pyrophosphate moiety (magenta and cyan sticks) of lipid II.

Nisin is synthesized as a precursor peptide that is processed into the 34-residue active form through the actions of a protease and modification enzymes (Buchman et al., 1988; Gross & Morell, 1971; Ra et al., 1999). A defining structural feature of nisin is the five intramolecular (methyl)lanthionine rings that are posttranslationally installed along the elongated peptide backbone (Fig. 3A) (Gross & Morell, 1971). A solution NMR structure of nisin in complex with lipid II revealed that the N-terminal (methyl)lanthionine rings in particular are important for nisin bioactivity as they mediate binding to lipid II (Fig. 3B) (Hsu et al., 2004). To determine the structure, a variant of lipid II with a triprenyl tail was used as the substrate to maximize solubility. The shortened tail was not expected to adversely affect nisin binding, and indeed, the nisin-lipid II structure showed that nisin recognizes MurNAc and only the first isoprene unit in the lipid tail as well as the pyrophosphate group linking the sugar and lipid. Upon binding to the substrate, nisin forms a pocket comprised of its first two residues, Ile1 and Dhb2, the N-terminal lanthionine ring A, and methyllanthionine ring B that encloses these regions of lipid II. Notably, hydrogen bonding between the pyrophosphate group of lipid II and the backbone amides, rather than specific side chains, of the nisin pocket drives the interaction.

The second step in the mode of action of nisin is generation of membrane pores. A structure of membrane-embedded nisin is unavailable, but the nisin-lipid II structure together with other *in vitro* and modeling studies suggest a mechanism for pore formation (Van Heusden et al., 2002; Wiedemann et al., 2004). Whereas the N-terminus of nisin binds to lipid II, it is the C-terminus of nisin that penetrates the cytoplasmic membrane. The N-and C-terminal regions are separated by a few residues that do not contribute to the rigid (methyl)lanthionine rings. These linear residues act as a flexible hinge (Fig. 3A) around which the C-terminus of nisin can pivot and insert perpendicularly into the phospholipid bilayer (Van Heusden et al., 2002). Therefore, lipid II positions nisin at the membrane and also appears to help stabilize the membrane-spanning nisin pores that form (‘t Hart et al., 2016). Interactions with membrane phospholipids may contribute to nisin bioactivity (Prince et al., 2016), but it is evident that lipid II plays a central role in the mode of action of nisin. A mechanism that relies on binding with the chemically conserved lipid II moiety explains how nisin is able to kill diverse Gram-positive pathogens. At the same time, this mechanism ensures targeted activity as nisin does not simply disrupt any lipid environment.

Other Gram-positive lanthipeptide antibiotics that inhibit bacterial cell wall synthesis appear to bind lipid II as well for bioactivity (McAuliffe et al., 2001). Two examples are provided by the lanthipeptides epidermin (Allgaier et al., 1985) and mutacin 1140 (Hillman et al., 1998), which are elongated nisin-like molecules that contain four thioether rings. Using purified cell wall substrates, epidermin was shown to form a complex with lipid II (Brötz et al., 1998). The interaction with lipid II is critical for the pore-forming activity of epidermin, as the peptide was only able to permeabilize synthetic liposomes when lipid II was incorporated in the liposomes (Brötz et al., 1998). Similar use of lipid-II containing liposomes support the binding interaction of mutacin 1140 to lipid II (Smith et al., 2008). Mersacidin is a small *Bacillus*-derived lanthipeptide that also contains four thioether rings (Chatterjee et al., 1992). However, unlike nisin, epidermin, and mutacin 1140, mersacidin adopts a more compact, globular shape (Bierbaum et al., 1995; Prasch et al., 1997). Early studies using macromolecular synthesis inhibition assays pinpointed peptidoglycan synthesis as the pathway that mersacidin targets (Brötz et al., 1995). Reconstitution of peptidoglycan synthesis with a cell-free system then showed that mersacidin inhibits peptidoglycan polymerization and leads to accumulation of lipid II (Brötz et al., 1997). In line with these observations, purified lipid II added in excess was found to antagonize mersacidin activity, increasing the minimal inhibitory concentration (MIC) of mersacidin against an indicator strain (Brötz et al., 1998). Consequently, it was proposed that mersacidin sequesters lipid II to inhibit peptidoglycan synthesis. A solution NMR study in membrane-mimetic dodecylphosphocholine (DPC) micelles later provided evidence of lipid II binding that is driven by electrostatic interactions with the E17 acidic side chain in mersacidin (Hsu et al., 2003). The GlcNAc sugar of lipid II may be involved in the binding interaction, as mersacidin has a much higher affinity for lipid II than lipid I, an intermediate that lacks GlcNAc (Fig. 2B) (Brötz et al., 1997, 1998).

Similar mechanistic and structural studies of actagardine, microbisporicin, and the two-component lacticin 3147 system show that these lanthipeptides likewise inhibit peptidoglycan synthesis. Some of these lanthipeptides also disrupt the cytoplasmic membrane. In the case of lacticin 3147, two peptides act synergistically to effect cell death (McAuliffe et al., 1998; Ryan et al., 1996). The first peptide, lacticin A1, has four (methyl)lanthionine rings and structurally resembles mersacidin. Lacticin A1 contains an equal number of acidic (D10 and E24) and basic (H23 and K30) residues. The second peptide, lacticin A2, has three (methyl)lanthionine rings and a more elongated structure (Martin et al., 2004). Lacticin A2 is cationic due to the presence of K24 and R27 at the C-terminus. It has been proposed that these two peptides recapitulate the two-step mechanism of nisin: lacticin A1 binds lipid II to associate with the cytoplasmic membrane and recruits lacticin A2, which inserts into the membrane (Wiedemann et al., 2006). Membrane insertion leads to formation of pores that release potassium ions, dissipating the membrane potential (Wiedemann et al., 2006). The absence of either peptide compromises lacticin activity (Wiedemann et al., 2006). Also important for lacticin bioactivity are its charged residues, in particular E24 of lacticin A1, which may mediate interaction with the cell membrane (Deegan et al., 2010). Interestingly, the location of charged residues in lacticin 3147 is quite conserved in other two-component lanthipeptides (Deegan et al., 2010). Actagardine (Arioli et al., 1976; Boakes et al., 2009; Coronelli et al., 1976; Parenti et al., 1976; Somma et al., 1977; Zimmermann & Jung, 1997) and microbisporicin (Castiglione et al., 2007, 2008; Münch et al., 2014; Vasile et al., 2012) are similar to lacticin in that they also have mersacidin-like domains that presumably underlie their peptidoglycan synthesis inhibiting activity. Microbisporicin, which is commercially known as NAI-107, appears to form a 1:1 or 2:1 complex with lipid II through a binding event that involves its N-terminal thioether rings and the lipid II pyrophosphate group as seen with nisin (Castiglione et al., 2007, 2008; Münch et al., 2014; Vasile et al., 2012). However, unlike nisin, microbisporicin treatment does not detectably induce the formation of membrane pores (Münch et al., 2014). A different lanthipeptide that has membrane-disrupting activity is bovicin HJ50 (Xiao et al., 2004). There is some evidence that bovicin HJ50 interacts with lipid II in the process of pore formation because the N-terminal fragment of nisin, which is known to bind lipid II, competitively inhibits bovicin activity (Zhang et al., 2014). Direct demonstration of this interaction is still needed.

Lipid II binding is evidently a key step in the action of many antibacterial lanthipeptides, but other lipid molecules can also serve as docking substrates. A well-known example is illustrated by the 19-residue cyclic lanthipeptide cinnamycin (Takemoto et al., 1984), also known as Ro09–0198, which binds the membrane phospholipid phosphatidylethanolamine (Choung, Kobayashi, Inoue, et al., 1988; Choung, Kobayashi, Takemoto, et al., 1988). Cinnamycin forms a compact structure as a result of three internal thioether crosslinks and an additional crosslink between the sidechains of the sixth and last residues, A6 and K19 (Fredenhagen et al., 1990; Kaletta et al., 1991; Kessler et al., 1987). Early investigations on the bioactivity of cinnamycin revealed that it is hemolytic against mammalian erythrocytes (Choung, Kobayashi, Inoue, et al., 1988), but its activity is not confined to targeting solely eukaryotic cell membranes. Cinnamycin also attacks the bacterial cell membrane through a conserved mechanism that relies on recognition of phosphatidylethanolamine lipids (O'Rourke et al., 2017; Widdick et al., 2003). Structurally, phosphatidylethanolamine is built on a glycerol backbone to which two fatty acids and an ethanolamine-phosphate head group are esterified. Binding studies with different types of lipids suggested that the identity of the polar head group—the presence of a free amino end and phosphate moiety—dictated whether cinnamycin could bind the lipid and permeabilize the membrane (Choung, Kobayashi, Inoue, et al., 1988; Choung, Kobayashi, Takemoto, et al., 1988). Subsequent NMR analysis and molecular dynamics simulation of the cinnamycin-phosphatidylethanolamine complex revealed key contacts underlying the interaction and explained how the peptide displays such selectivity toward the lipid (Hosoda et al., 1996; Vestergaard et al., 2019; Wakamatsu et al., 1990). Cinnamycin forms a snug pocket that surrounds the phosphatidylethanolamine polar head group in a 1:1 complex, with the acyl chains sticking out from the peptide (Hosoda et al., 1996; Vestergaard et al., 2019; Wakamatsu et al., 1990). The primary amine of the lipid head group contacts the backbone carbonyls of cinnamycin and the hydroxylated D15 residue through hydrogen bonding. A network of hydrogen bonds between the backbone of cinnamycin residues F10-Abu11-F12-V13 and the phosphate group of phosphatidylethanolamine contributes to further stabilization of the complex (Vestergaard et al., 2019). How cinnamycin engages the lipid presumably affects its capacity to permeabilize the membrane (Vestergaard et al., 2019).

These lanthipeptide studies point to key features of this class of RiPPs. Many lanthipeptides are pore-forming agents that seemingly have evolved to appropriate lipid II as an anchor for attachment to the cell membrane (McAuliffe et al., 2001). Peptide binding to lipid II would also adversely impact cell wall synthesis because the enzymes that make new peptidoglycan material can no longer physically access this key substrate. The steady-state levels of Lipid II are after all normally low in cells (van Heijenoort, 2007; Bouhss et al., 2008). It is worth noting that the lanthipeptides discussed herein can recognize a different region of lipid II from glycopeptides such as vancomycin, which instead binds the terminal d-Ala-d-Ala residues of the lipid II stem peptide (Perkins, 1969). In fact, competitive lipid II binding assays helped reveal that the mode of action of mersacidin, for example, is distinct from those of glycopeptides (Brötz et al., 1998). Such unique mechanisms may be informative for developing combinatorial antibiotics. Another theme that emerges from comparison of these lanthipeptides is the structural conservation of domains that bind lipid II and how homology-based searches can hone in on mechanisms of action.

### Polytheonamide Toxins that form Membrane Channels

Some of the largest and most heavily modified RiPPs discovered to date are the polytheonamides, a class of RiPPs that exhibits ion channel activity. Polytheonamides are marine sponge-derived founding members of the proteusin class of RiPPs, a moniker fittingly inspired by the Greek sea god Proteus (Freeman et al., 2012). Polytheonamides are potent cytotoxic agents against mammalian cells whose mode of action involves ion channel formation (Iwamoto et al., 2010). In 1994, Hamada et al. isolated two polytheonamides, A and B, from the marine sponge *Theonella swinhoei* and preliminarily analyzed their primary sequences (Hamada et al., 1994a; Hamada et al., 1994b). A revised characterization of the sequences revealed that the polytheonamides are 49-residue linear peptides that extensively contain unusual amino acids in the d-configuration (Hamada et al., 2005). The A and B peptides differ only in the stereochemistry of the sulfoxide modification at residue 44 (Hamada et al., 2005). Given the high prevalence of nonproteinogenic amino acids, for a long time the polytheonamides were thought to be nonribosomal peptides (Hamada et al., 2005). However, the enzymatic machinery responsible for their synthesis remained elusive for almost two decades. In 2012, a seminal report unveiled the polytheonamide BGC (Freeman et al., 2012). The relatively few modification enzymes encoded within the polytheonamide gene cluster could account for most of the 40+ posttranslational modifications observed (Freeman et al., 2017). Identification of the gene cluster answered a longstanding question. That is, by finding the structural gene which specifies the precursor peptides, the authors demonstrated that polytheonamides A and B were in fact ribosomal peptides. Moreover, the operon-like organization of the gene cluster suggested a bacterial origin. The polytheonamide producer was subsequently identified as an uncultivated filamentous bacterial symbiont from the genus *Entotheonella* that lives in marine sponges (Wilson et al., 2014).

**Fig. 4.**
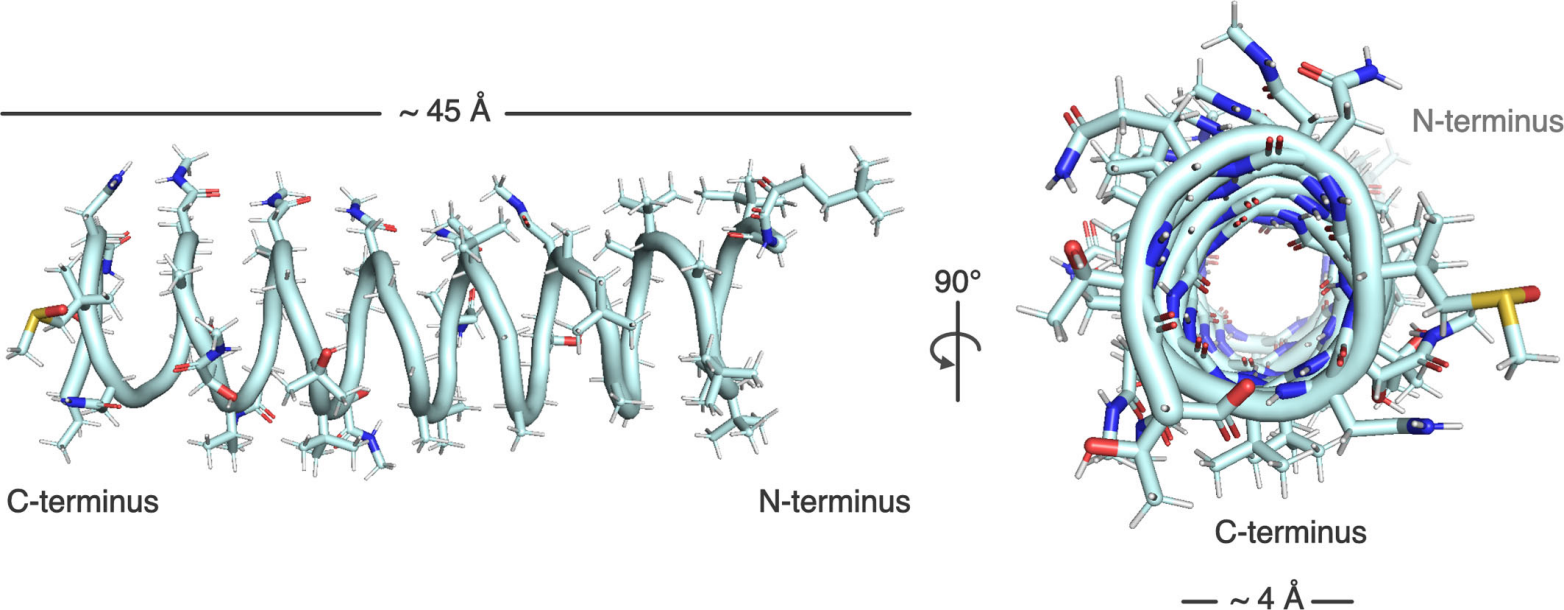
The β-spiral structure of polytheonamide B in organic solvent to mimic the membrane environment (PDB file 2RQO). Left: View showing the length of polytheonamide B to be roughly the same thickness as a plasma membrane. Note the density of polar residues at the C-terminus of the structure. Right: The interior of polytheonamide B is polar, allowing for the passage of cations such as potassium. Polytheonamide B is a potent cytotoxin, functioning as an ion channel.

That the polytheonamides are capable of forming ion channels which induce cytotoxicity can be attributed to their three-dimensional structure. Not only are the polytheonamides replete of unusual amino acids, many of the amino acids are hydrophobic in nature. The N-terminus of the peptides is also blocked by a *tert*-butylated *N*-acyl group (Hamada et al., 2005). The overall hydrophobicity of the peptide implies membrane localization. Another striking feature of polytheonamides is the alternating pattern of l- and d-amino acids along the length of the peptides (Hamada et al., 2005). This pattern is a signature of the nonribosomal gramicidin peptides, which form membrane ion channels that disturb the cellular balance of ions (Kelkar & Chattopadhyay, 2007). Polytheonamide B was likewise found to structurally organize as a β-helix resembling a spring or corkscrew (Hamada et al., 2010). The solution NMR structure in membrane-mimetic methanol: chloroform (1:1) solvent indicated that the polytheonamide B helix is approximately 45 Å in length and the diameter of the pore is 4 Å (Fig. 4). These physical dimensions have two important functional implications. The average thickness of the plasma membrane has been estimated to be 30–40 Å (Andersen & Koeppe, 2007; Mitra et al., 2004; van Meer et al., 2008); as such, a single polytheonamide helix can span the entire phospholipid bilayer to form an ion channel (Hamada et al., 2010). Second, the ionic radius of a potassium ion—a demonstrated cargo of polytheonamide channels (Hamada et al., 2010; Iwamoto et al., 2014)—is 1.33 Å (Huang et al., 1978), which means that the ions can readily be conveyed through the interior of the helix assuming the absence of competing cargo. Besides potassium, other monovalent cations also demonstrate the ability to traverse the polytheonamide channel with the experimentally tested selectivity of H+ > Cs+ > Rb+ > K+ > Na+ (Hamada et al., 2010; Oiki et al., 1997). As expected for an ion channel, the polytheonamide B helix has a hydrophilic core (Hamada et al., 2010). Another feature of the peptide is that its N-terminus is more hydrophobic than its C-terminus, which led to the hypothesis that the N-terminus initially punctures the membrane (Hamada et al., 2010; Shinohara et al., 2012). In addition to forming plasma membrane ion channels, polytheonamides may exert secondary activity in other cellular compartments. According to a recent report, polytheonamide B dissipates the pH gradient of lysosomes purportedly through similar pore-forming activity (Hayata et al., 2018). Mechanisms to maintain ion gradients across the cell membrane are essential to life. Protein channels ferry ions across the impermeable cell membrane, and importantly, are subject to stringent regulation. By dysregulating the flow of ions, polytheonamide toxins effectively disrupt cellular ion homeostasis and membrane potential.

### Membrane-Active Bacteriocin RiPPs

Another widespread class of bacterial ribosomal peptides with membrane-targeting activity is the bacteriocins. Bacteriocins are not classified according to a unique posttranslational modification, but rather are broadly defined as antimicrobial proteinaceous substances. As such, bacteriocins come in many different varieties, harboring diverse chemical modifications and having a broad spectrum of activity. Nisin and the other lanthipeptides discussed above constitute a subset of bacteriocins. Like nisin, many bacteriocins are used as natural, safe preservatives to kill foodborne pathogens and minimize food spoilage (Drider et al., 2006). Some bacteriocins, such as lactococcin G (Kjos et al., 2014; Nissen-Meyer et al., 1992) and plantaricin JK (Anderssen et al., 1998; Oppegård et al., 2016), consist of two peptide components and lack posttranslational modifications. Over the past century, activity-driven assays have unearthed hundreds of bacteriocins. For instance, lactic acid bacteria alone harbor more than 230 putative bacteriocin BGCs and are prolific producers of these RiPPs (Alvarez-Sieiro et al., 2016). The biosynthesis and activities of bacteriocins have been extensively reviewed (Arnison et al., 2013; Li & Rebuffat, 2020). This section will survey representative peptides from a few different classes of membrane-active, posttranslationally modified bacteriocins for which structural data exist. The mechanistic studies that revealed how peptide form dictates function will be the subject of focus.

**Fig. 5.**
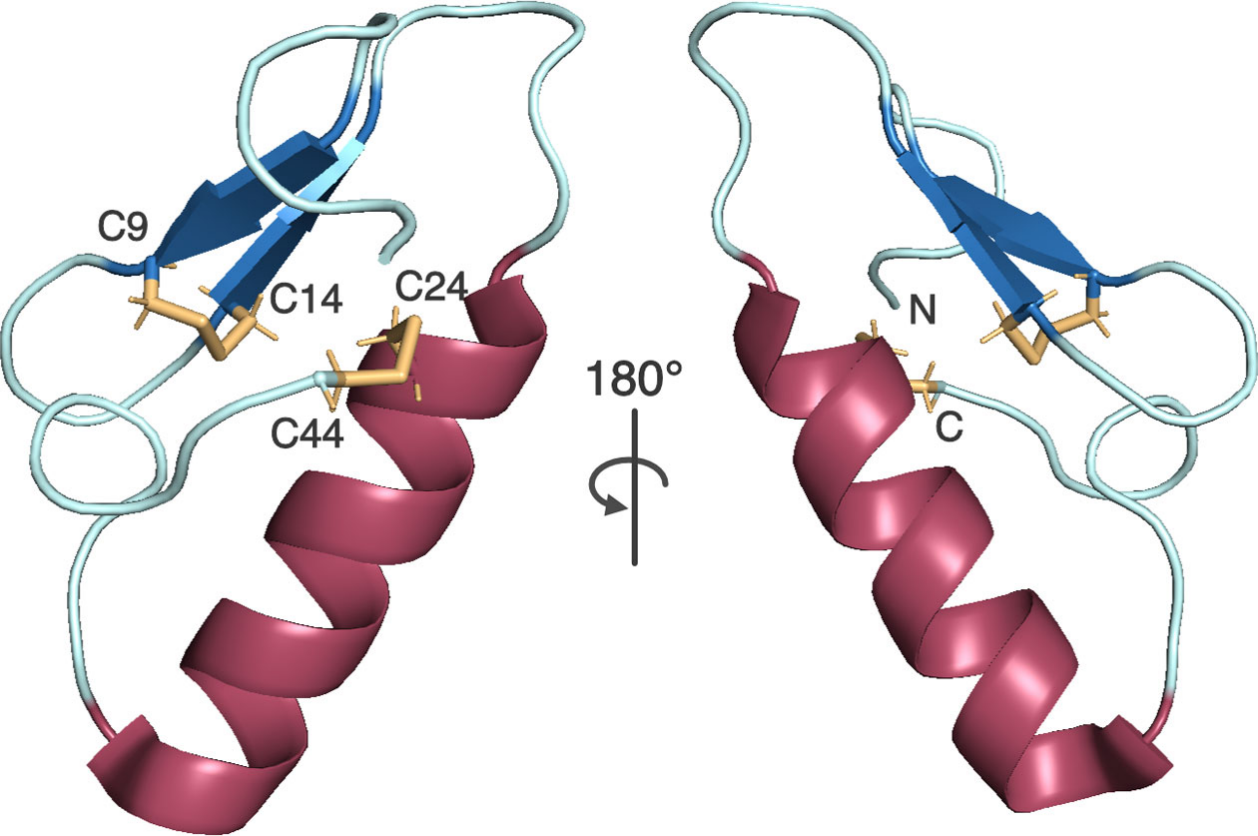
Structure of a synthetic analog of pediocin PA-1, a membrane-active bacteriocin. This compact structure is formed via two disulfide bonds, which are highlighted in orange. The N-terminal region of pediocin PA-1 is cationic, allowing for electrostatic interactions with bacterial membrane lipid head groups, while its C-terminal region is hydrophobic, allowing for pore formation in membranes. Figure drawn from PDB file 5UKZ.

Pediocin-like bacteriocins are defined by a conserved, minimally modified tripartite structure consisting of an N-terminal antiparallel β-sheet, an intervening α-helix, and an unstructured C-terminal tail (Balandin et al., 2019; Fimland et al., 2005; Ríos Colombo et al., 2018). A prototypical example of this RiPP class is pediocin PA-1, also known as pediocin AcH (Bhunia et al., 1988; Gonzalez & Kunka, 1987; Henderson et al., 1992; Motlagh et al., 1992). Pediocin PA-1 has only a single type of posttranslational modification in the form of two disulfide bridges linking C9–C14 at the N-terminus and C24–C44 at the C-terminus (Fig. 5) (Henderson et al., 1992). The N-terminal disulfide bridge acts as a staple to stabilize the β-sheet and is found in almost all pediocin-like bacteriocins (Bédard et al., 2018). Another conserved N-terminal motif found in pediocin PA-1 and similar peptides is the pediocin box, which describes the sequence YGNG(V/L) that when mutated leads to reduction of pediocin antimicrobial activity (Miller et al., 1998). Mutations of the disulfide bond-forming cysteines similarly reduces pediocin activity (Bédard et al., 2018; Miller et al., 1998). Pediocin PA-1 was found to adsorb to Gram-positive cells in a process that likely involved electrostatic interactions, providing initial evidence of membrane-targeting activity (Bhunia et al., 1991). Curiously, pediocin PA-1 could adsorb to both pediocin-resistant and susceptible Gram-positive strains. This observation led to the proposal that a cell-surface receptor that is the specific docking site of pediocin PA-1 is present only in susceptible cells. Adsorption of pediocin PA-1 to susceptible cells caused cells to lyse or cease growing; release of potassium ions indicative of membrane potential collapse was also observed. Efflux and transport assays, transmembrane potential measurements, and binding studies provided additional evidence that pediocin PA-1 permeabilizes the bacterial cytoplasmic membrane (Chen, Ludescher, et al., 1997; Chen, Shapira, et al., 1997; Chikindas et al., 1993). Specifically, the cationic N-terminus of pediocin PA-1 alone can bind membrane vesicles but the more hydrophobic C-terminus is required for pore formation (Chen, Ludescher, et al., 1997). The C-terminus also predominantly determines target cell specificity (Johnsen et al., 2005). However, whether pediocin PA-1 required a membrane protein receptor for activity was debatable because it could release carboxyfluorescein from vesicles composed entirely of synthetic phospholipids (Chen, Shapira, et al., 1997). The σ^54^-regulated mannose phosphotransferase (Man-PTS) system has been proposed as the membrane receptor for pediocin-like bacteriocins (Dalet et al., 2000, 2001; Ramnath et al., 2000; Robichon et al., 1997). Yet, the relative contribution of this putative receptor toward peptide-membrane interaction is unclear. How the different regions of the multisubunit Man-PTS system would engage with the peptide is also poorly understood. Interestingly, the Man-PTS system is apparently required for the bioactivity of other structurally distinct bacteriocin RiPPs and may serve as a membrane receptor in these cases as well. The nonpediocin-like garvicin A/B/C peptides that target a Man-PTS subunit provide one such example (Tymoszewska et al., 2018).

**Fig. 6.**
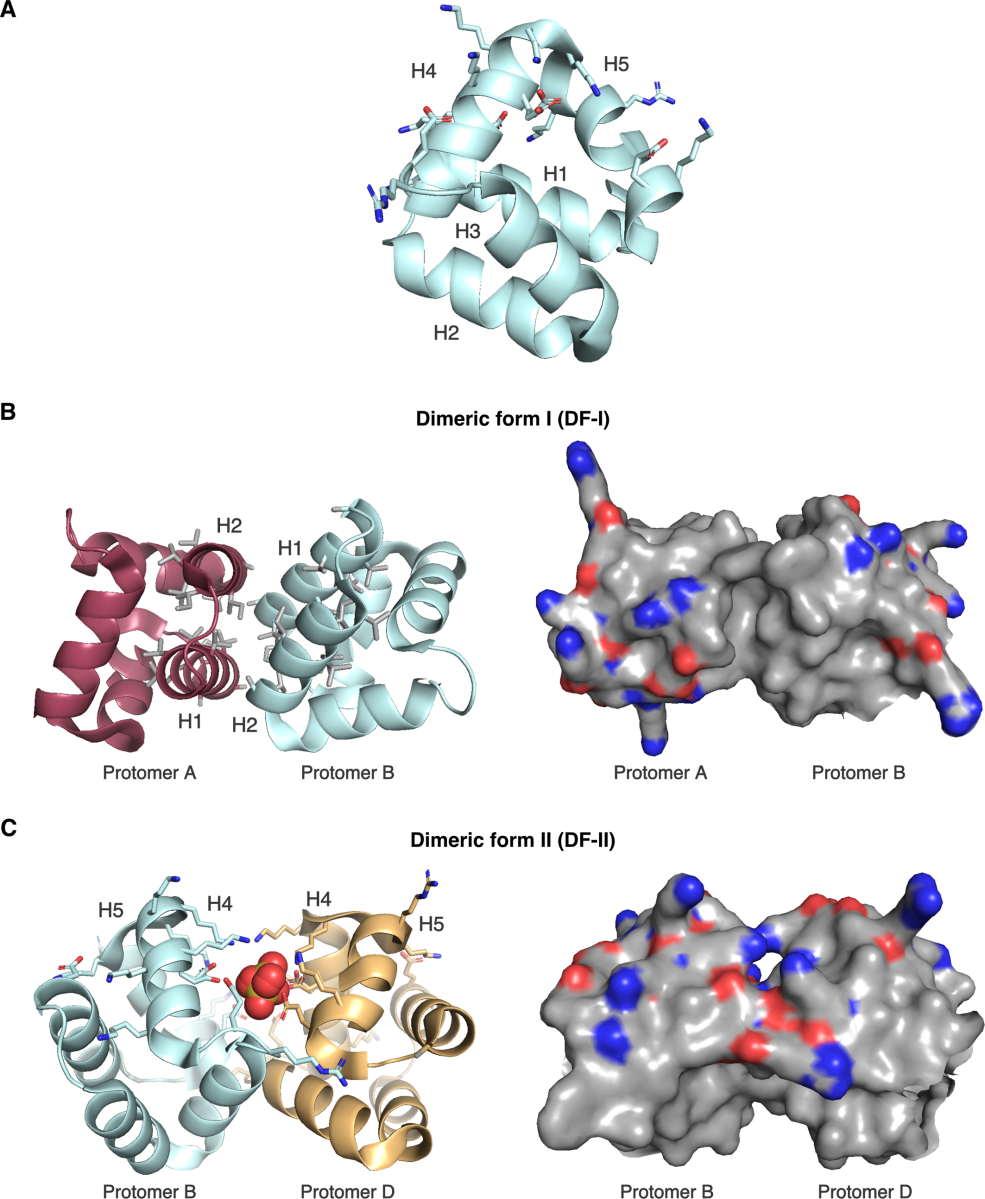
Structure of the membrane-active bacteriocin enterocin AS-48. (A) Enterocin AS-48 is cyclized between its N- and C-termini and highly helical. Sidechains of negatively and positively charged amino acids are clustered together in helices 4 and 5 and are shown as sticks (PDB file 1O82). (B and C) Two distinct dimeric forms of enterocin AS-48 are found within the crystal structure. Dimeric form I (DF-I) shows the hydrophobic side chains of helices 1 and 2 in gray. Dimeric form II (DF-II) includes a sulfate ion at the protomer–protomer interface.

The head-to-tail circularized bacteriocins are another group of membrane-active RiPPs that lack extensive posttranslational modifications. The founding member of this group is enterocin AS-48, a 70-residue peptide whose N- and C-termini are connected through a peptide bond (Burgos et al., 2014; Gálvez et al., 1986, 1989; Martinez-Bueno et al., 1994; Samyn et al., 1994). Enterocin AS-48 has broad-spectrum antimicrobial activity against several Gram-positive pathogens and to a lesser extent some Gram-negative organisms (Gálvez et al., 1986). Treatment of sensitive cells with enterocin AS-48 led to collapse of cytoplasmic membrane potential and formation of pores through which solutes could leak (Gálvez et al., 1991). As enterocin AS-48 can permeabilize artificial lipid vesicles, its interaction with the membrane may occur independently of a protein receptor (Gálvez et al., 1991). Solution NMR and crystal structures of enterocin AS-48 provided a model for how the peptide acts on the cell membrane (González et al., 2000; Sánchez-Barrena et al., 2003). The peptide folds into a compact globular shape that consists of five α-helices arranged in such a way that their hydrophobic side chains are directed toward the core of the 3D structure (González et al., 2000). The α-helices and the intervening loops are stabilized by a network of hydrogen bonds. Due to the high number of lysines present in the sequence, enterocin AS-48 has a net positive charge. However, the charged residues cluster within helices α4 and α5, the latter of which contains the head-to-tail junction of the cyclized peptide (Fig. 6A). One side of the enterocin AS-48 three-dimensional surface is more cationic as a consequence of this asymmetrical distribution of charged residues. The initial stages of peptide-membrane engagement may rely on electrostatic interactions between this cationic surface and the anionic head groups of acidic phospholipids that are enriched in bacterial cell membranes (González et al., 2000). Once enterocin AS-48 attaches with the membrane, rearrangement of the peptide to expose hydrophobic residues may facilitate membrane insertion (Sánchez-Barrena et al., 2003). Crystal structures of enterocin AS-48 in two different oligomeric conformations differing in the relative positions of the hydrophobic and hydrophilic α-helices support this rearrangement-dependent mechanism (Fig. 6B, C) (Sánchez-Barrena et al., 2003).

Gram-negative bacteria also produce modified bacteriocin RiPPs that target the cytoplasmic membrane of closely related species. An example of a membrane-active, Gram-negative bacteriocin is microcin E492, which was isolated from *Klebsiella pneumoniae* strain RYC492 and showed broad activity against members of the *Enterobacteriaceae* family (de Lorenzo, 1984; Lagos et al., 2009). Microcin E492 is a low-molecular weight peptide as is typical for the microcin family of RiPPs, but its production is specified by a BGC that resides on the chromosome instead of a plasmid as for many other microcins. Identification (Wilkens et al., 1997) and sequencing (Lagos et al., 2008) of the BGC revealed several potential modification enzymes, whose roles were later revealed to be installation of a C-glucosylated linear trimer of *N*-(2,3 dihydroxybenzoyl)-l-serine (DHBS) to the C-terminus of the precursor peptide (Vassiliadis et al., 2007). Microcin E492 exists in both the modified and unmodified form, but it is the modified form that exhibits more pronounced antimicrobial activity (Thomas et al., 2004). The observed difference in activity has been attributed to the relative affinities of the two peptide forms for the outer membrane transporter that delivers microcin E492 to its site of action at the cytoplasmic membrane (Destoumieux-Garzón et al., 2006; Thomas et al., 2004). In fact, the C-glucosylated DHBS unit of the modified peptide structurally resembles the catecholate siderophore salmochelin (Thomas et al., 2004; Vassiliadis et al., 2007). Siderophore uptake occurs through TonB-dependent transporters (TBDTs) (Noinaj et al., 2010). Therefore, it has been proposed that the siderophore-like appendage of microcin E492 directs peptide transport across the outer membrane through a TBDT. Consistent with this role, it was shown that TonB and the FepA, Fiu, and Cir outer membrane receptors of target cells are required for microcin E492 bioactivity (Patzer et al., 2003; Thomas et al., 2004). Perhaps also consistent with TonB-dependent transport of microcin E492, another catecholate-type siderophore, enterochelin, was found to inhibit bioactivity possibly through competition for the same transporter (Orellana & Lagos, 1996). Mechanistic and structural information on microcin E492-receptor interaction is still unknown. Once it has crossed the outer membrane barrier, microcin E492 is thought to permeabilize the cytoplasmic membrane (De Lorenzo & Pugsley, 1985). Membrane insertion releases membrane potential-sensitive probes from preloaded cells, indicating depolarization of the membrane (De Lorenzo & Pugsley, 1985; Destoumieux-Garzón et al., 2003). In addition to antibacterial activity, microcin E492 has been reported to induce apoptosis of some human cancer cell lines, although whether this secondary activity is related to its pore-forming ability is unclear (Hetz et al., 2002). Going forward, how microcin E492 engages with the cytoplasmic membrane and how this process may involve inner membrane proteins remain to be seen (Bieler et al., 2006). It has also been reported that microcin E492 forms amyloid-like fibrils that preclude activity (Bieler et al., 2005). What domains of microcin E492 are necessary and sufficient for bioactivity and the route of conversion to a different oligomeric state are potential future areas of investigation.

**Fig. 7.**
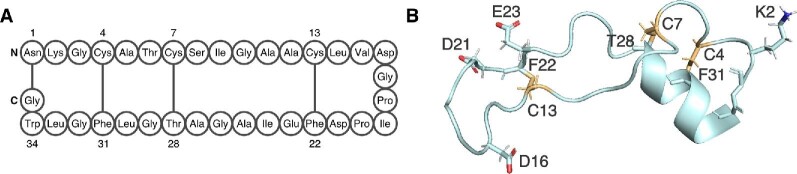
The structure of sactipeptide subtilosin A. (A) The 35 aa subtilosin A is cyclized via a head-to-tail peptide bond and three unusual thioether linkages between the side chain of cysteine and the α-carbon of a distal amino acid. (B) The solution structure of subtilosin A in methanol (PDB file 1PXQ) shows a compact hairpin-like structure. Acidic (D16, D21, E23) and basic (K2) residues are concentrated at different ends of the folded peptide. Residues participating in thioether linkages are shown as gold sticks.

Sactipeptides are a growing class of bacteriocin RiPPs that are defined by unusual intramolecular covalent linkages between the side chain sulfur of cysteine residues and the α-carbon of other residues. The most well-characterized sactipeptide is sutilosin A, which was first isolated from the common laboratory strain *Bacillus subtilis* 168 (Babasaki et al., 1985) and subsequently from other *Bacillus* species (Sutyak et al., 2008; Zheng et al., 1999). Subtilosin A has bactericidal activity against Gram-positive pathogens (Babasaki et al., 1985) and reportedly spermicidal activity as well (Silkin et al., 2008). Four intramolecular linkages contribute to the semirigid, cyclic nature of subtilosin A (Babasaki et al., 1985; Kawulka et al., 2003, 2004; Marx et al., 2001). In addition to three thioether bridges that connect C4 with F31, C7 with T28, and C13 with F22, an amide bond between N1 and G35 links the N- and C-terminus of the peptide (Fig. 7A) (Kawulka et al., 2003, 2004). A solution NMR structure of subtilosin A resembled a bent hairpin that forms a shallow basin (Fig. 7B) (Kawulka et al., 2003). The two sides of the hairpin are held close together by the thioether linkages and the side chains of the backbone point outward from the center of the hairpin loop (Kawulka et al., 2003, 2004). Subtilosin A has many hydrophobic residues, but there are also three acidic residues that are concentrated on the loop end of the folded peptide and a basic residue present on the opposite end. This separation of charge is believed to impact how subtilosin A targets the cytoplasmic membrane of sensitive cells (Thennarasu et al., 2005). Subtilosin A bound and permeabilized unilamellar vesicles composed of synthetic phospholipids (Thennarasu et al., 2005). The model proposed for binding involves insertion of the loop-distal end of subtilosin A into the lipid bilayer first (Thennarasu et al., 2005). This end contains the single basic residue, K2, which may interact with the negatively charged phosphate headgroup and a large, hydrophobic tryptophan residue, W34, which may perturb the lipid bilayer (Thennarasu et al., 2005). Release of membrane potential- and pH-sensitive probes from preloaded cells suggest that partial insertion of subtilosin A leads to ATP efflux and depletion of the transmembrane ion gradient (Noll et al., 2011).

Similarly, the two-component sactipeptide thuricin CD, which has nanomolar potency against *Clostridium* species, also attacks the bacterial cytoplasmic membrane (Mathur et al., 2017; Rea et al., 2010). Thuricin CD is composed of two structurally related peptides, α and β, that share 39.6 per cent sequence identity and act synergistically (Rea et al., 2010). The residues at positions 21, 25, and 28 in both peptides are modified through α-carbon-cysteine sulfur linkages. Neither peptide has the N-to-C amide linkage present in subtilosin A, but thuricin-α and thuricin-β each forms a folded hairpin structure with the backbone side chains pointed outward similar to subtilosin A (Sit et al., 2011). The solution NMR structure of thuricin CD also showed that the surfaces of the constituent peptides are highly hydrophobic (Sit et al., 2011). Upon thuricin CD challenge, sensitive cells undergo membrane depolarization and cell lysis (Mathur et al., 2017). A more recent example of a sactipeptide with proposed membrane-targeting activity is thuricin Z (Mo et al., 2019). Thuricin Z has four α-carbon to cysteine sulfur linkages installed at residues D26-C16, T30-C12, Y34-C8, and H38-C4, highlighting a degree of periodicity in the modified sites (Mo et al., 2019). To our knowledge, the three-dimensional structure of thuricin Z has not been solved. However, like subtilosin A, thuricin Z also permeabilizes bacterial membrane-mimicking unilamellar vesicles (Mo et al., 2019; Thennarasu et al., 2005).

Bacteriocin RiPPs are extremely diverse in structure and tailoring modifications, but a striking number appear to act on the same target, the bacterial cytoplasmic membrane. Binding studies with purified peptides and model membrane vesicles have provided insights into the chemical requirements for interaction and pore formation. Electrostatic interactions for one appear to play a strong role. Bacterial cell membranes are rich in anionic phospholipids (Sohlenkamp & Geiger, 2016). Different bacteria have distinctive membrane lipid content and even within the same organism, the membrane lipid content can adapt depending on the growth environment (Sohlenkamp & Geiger, 2016). The precise molecular interactions between bacteriocin RiPPs and diverse membrane structures remain to be dissected. Furthermore, it is worth considering how in reality the crowded physiological environment of a cell membrane would impact peptide binding. Sometimes, a concentration of bacteriocin higher than its MIC was required to observe membrane permeabilization *in vitro* (Thennarasu et al., 2005). This difference may reflect the mode by which the peptide engages the membrane. Even if a peptide can bind synthetic lipid-only vesicles *in vitro*, one of the many membrane-embedded proteins may promote binding *in cellulo*. Understanding these interactions will explain and enable prediction of spectrum of activity.

### Two Recently Discovered Cell Envelope-Targeting RiPPs

Aside from lanthipeptides, a member of the lasso peptide class of RiPPs has been reported to also inhibit bacterial cell wall synthesis by targeting lipid II. Siamycin-I is a 21-residue peptide that folds into a three-dimensional lasso structure containing a ring, loop, and tail regions (Detlefsen et al., 1995). An isopeptide bond between the N-terminal cysteine and side chain of D9 produces a nine-membered ring through which the remaining residues are threaded. Two disulfide bridges connecting the ring with the loop above and tail below further rigidify the lasso fold (Maksimov et al., 2012). The lipid II-targeting activity of siamycin-I was only recently reported (Daniel-Ivad et al., 2017; Tan et al., 2019), but siamycin-I had been shown to possess other bioactivities since its initial discovery more than two decades ago (Chokekijchai et al., 1995; Tsunakawa et al., 1995). Siamycin-I inhibits the human immunodeficiency virus (Chokekijchai et al., 1995; Lin et al., 1996; Tsunakawa et al., 1995), myosin light chain kinase involved in smooth muscle contraction (Yano et al., 1996), and ATP-dependent enzymes (Ma et al., 2011). Siamycin-I was rediscovered in a screen for cryptic secondary metabolites that are regulated by a two-component system (Daniel-Ivad et al., 2017). Expressing a constitutively active pleiotropic regulator triggered the production of this lasso peptide in multiple *Streptomyces* species (Daniel-Ivad et al., 2017). Several lines of evidence thereafter suggested siamycin-I inhibits bacterial cell wall synthesis. Treatment of sensitive cells with siamycin-I phenocopied treatment with the cell wall-targeting antibiotics vancomycin, bacitracin, and nisin (Daniel-Ivad et al., 2017). Mutations that conferred siamycin-I resistance mapped to the WalKR genes, which is a signal transduction pathway that controls cell wall metabolism (Tan et al., 2019). Finally, biochemical reconstitution with defined cell wall substrates demonstrated that siamycin-I blocks the conversion of polyprenyl pyrophosphate-linked substrates (Tan et al., 2019). Thus, it was concluded that siamycin-I binds lipid II through recognition of the conserved pyrophosphate motif similar to nisin (Tan et al., 2019). Unlike nisin however, siamycin-I treatment does not lead to formation of membrane pores (Tan et al., 2019). Siamycin-I is active against Gram-positive but not Gram-negative bacteria possibly due to the presence of an outer membrane barrier in the latter group (Daniel-Ivad et al., 2017; Tsunakawa et al., 1995). Nonetheless, the extent to which siamycin-I inhibits different Gram-positive bacteria does vary (Daniel-Ivad et al., 2017).

**Fig. 8.**
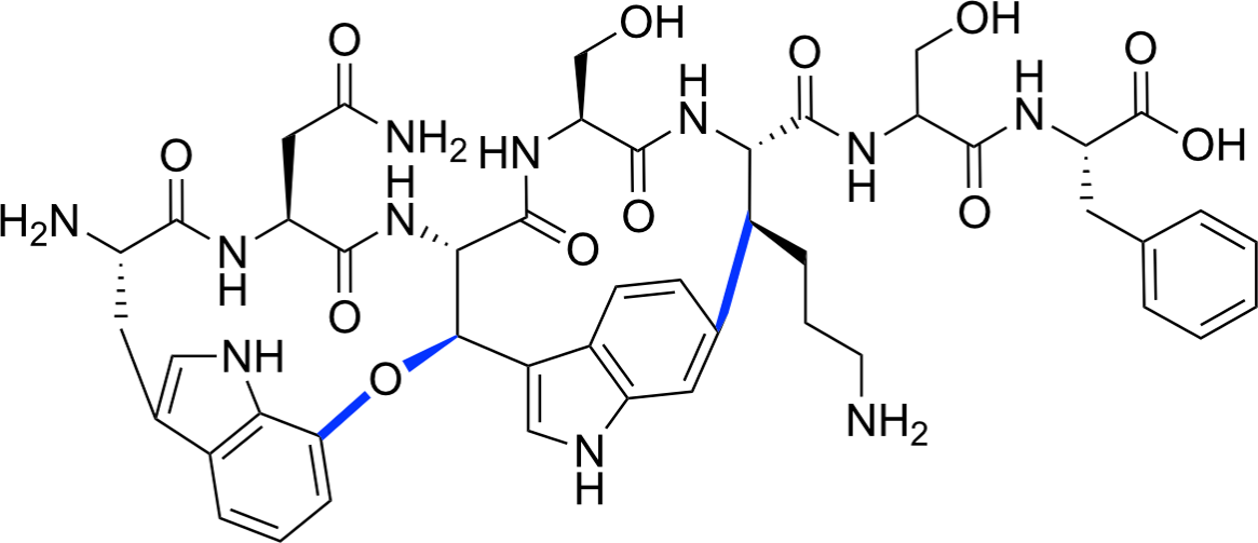
Chemical structure of the heptapeptide RiPP darobactin. Linkages between sidechains are colored in blue.

Darobactin is the founding member of a new class of RiPPs that are defined by the presence of strained cyclophane rings (Imai et al., 2019; Nguyen et al., 2020). Darobactin is a modified heptapeptide that contains two macrocycles formed through an aromatic-aliphatic ether linkage between the W1 side chain and W3 β-carbon and a carbon-carbon bond between the W3 side chain and K5 β-carbon (Fig. 8) (Imai et al., 2019). Imai et al. isolated darobactin by screening concentrated extracts from bacterial cultures of nematode gut symbionts for antimicrobial activity (Imai et al., 2019). Darobactin has high therapeutic potential because it showed broad-spectrum activity against Gram-negative bacteria and low cytotoxicity against human cell lines and commensals (Imai et al., 2019). An early clue in determining the mechanism of action was the observation that darobactin treatment decreased the abundance of outer membrane proteins while increasing the abundance of periplasmic chaperones (Imai et al., 2019). Spontaneous suppressor mutants that were resistant to darobactin mapped to BamA (Imai et al., 2019). The BamABCDE complex, or β-barrel assembly machinery (BAM), is an essential outer membrane complex that folds and inserts proteins into the outer membrane (Fig. 2B) (Bakelar et al., 2016; Rigel & Silhavy, 2012; Tomasek et al., 2020; Wu et al., 2005). The BamA subunit is a β-barrel protein that templates the folding of nascent β-barrels (Tomasek et al., 2020). BamA exists in different conformational states and switches from the lateral gate-closed to gate-opened state to accommodate entry of the unfolded substrate for folding (Hartmann et al., 2018; Tomasek et al., 2020). *In vitro* reconstitution of BAM in lipid nanodiscs showed that darobactin directly inhibited BAM activity and that activity was dependent on the modified peptide scaffold as a linear variant was inactive (Imai et al., 2019). Isothermal titration calorimetry and solution NMR experiments confirmed that darobactin bound BamA and stabilized its gate-closed conformation, thereby preventing opening of the lateral gate for substrate entry and folding (Imai et al., 2019). A closed BamA gate would lead to accumulation of unfolded proteins in the periplasm, which explains the observed increase in periplasmic chaperones (Imai et al., 2019). High-priority ESKAPE pathogens, many of which are Gram-negatives, have developed resistance to clinically used antibiotics (CDC, 2019). Antibiotics that are effective against Gram-negative pathogens are thus increasingly needed. A major impediment in developing these antibiotics is that the outer membrane provides a barrier that prevents accessibility to druggable targets. Cell-surface components, and in particular outer membrane components such as BamA, are suitable targets that circumvent the issue of outer membrane permeability. Notably, mutations in outer membrane components that confer resistance to a drug *in vitro* may simultaneously impair virulence *in vivo*. For instance, darobactin-resistant *E. coli* mutants that have acquired mutations in BamA have attenuated virulence in mice (Imai et al., 2019). This duality means that darobactin would be even more effective as a drug because resistant strains that arise are likely to also be less virulent.

## Transcription-Inhibiting RiPPs

Transcription is an essential step of gene expression where a specific coding sequence of DNA is converted to a corresponding messenger RNA by the enzyme RNA polymerase. The essentiality of this process makes RNA polymerase a promising drug target. In fact, various RiPPs, including many members in the family of lasso peptides and the cyclic peptide α-amanitin, have been unveiled to be RNA polymerase inhibitors through either crystallography or *in vitro* inhibition assays (Braffman et al., 2019; Cheung-Lee, Parry, Cartagena, et al., 2019; Cheung-Lee, Parry, Zong, et al., 2020; Mukhopadhyay et al., 2004; Solbiati et al., 1999).

**Fig. 9.**
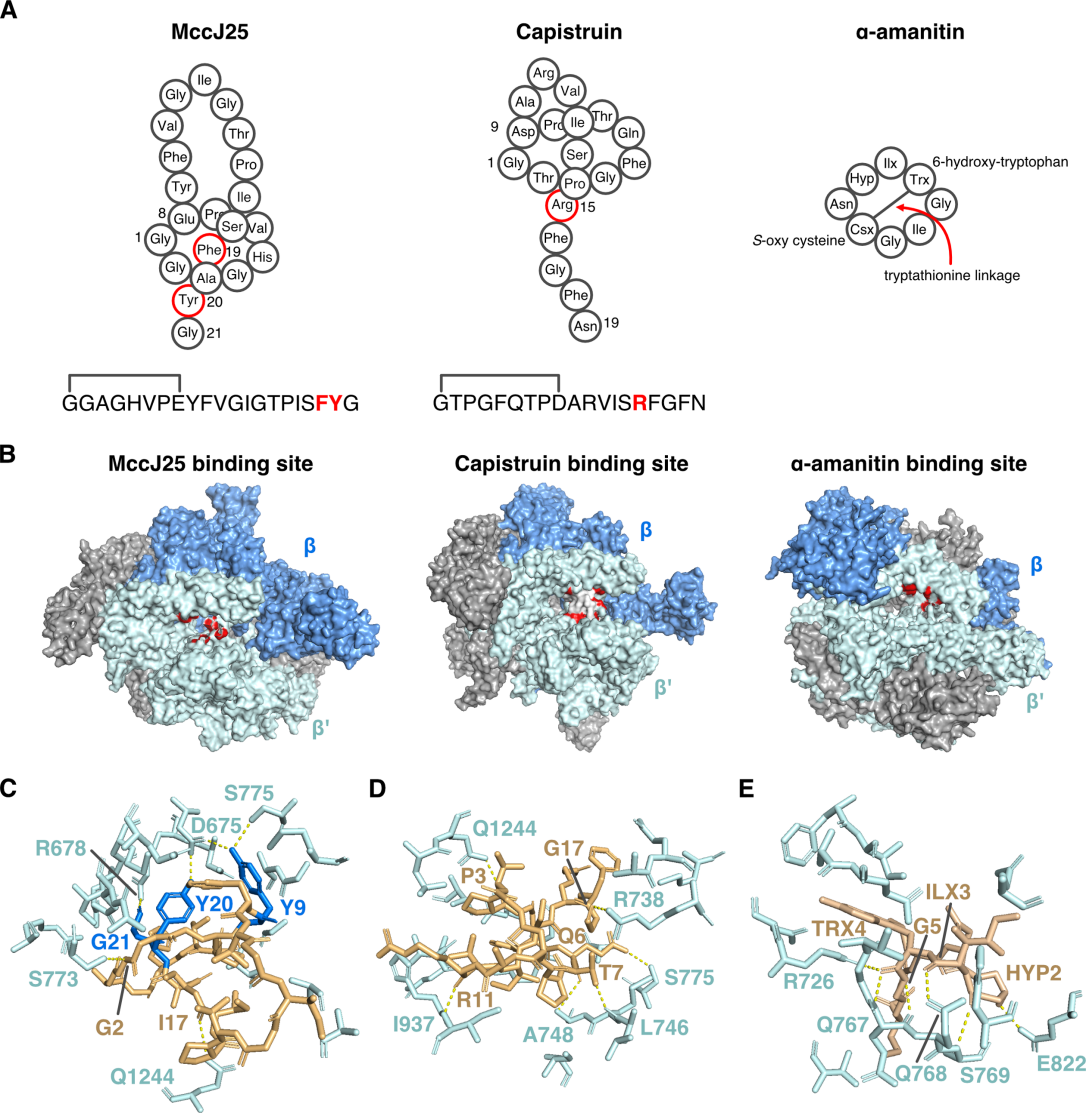
RiPP inhibitors of RNA polymerase (RNAP). (A) Schematics of microcin J25 (MccJ25), capistruin, and α-amanitin. ILX: 4,5-dihydroxyisoleucine, TRX: 6-hydroxytryptophan, CSX: S-oxy cysteine, HYP: HYP: 4-hydroxyproline. Note the tryptathionine linkage between 6-hydroxytryptophan and S-oxy cysteine. (B) From left to right, crystal structures of *E. coli* RNAP showing the binding site of MccJ25, *E. coli* RNAP showing the binding site of capistruin, and yeast RNA polymerase II (Pol II) showing the binding site for α-amanitin. Residues in RNAP or Pol II that directly contact these RiPPs are colored red. (C) Zoomed-in view of MccJ25 (gold/blue) bound to RNAP (cyan). Key amino acids for this interaction are labeled. Dashed lines are H-bonds (yellow). (D) As in part C, but for the RNAP–capistruin interaction. (E) Zoomed-in view of the α-amanitin-Pol II binding site with key interacting residues highlighted. HYP: hydroxyproline, ILX: dihydroxyisoleucine, TRX: hydroxytryptophan. Drawn from PDB files 6N60 (MccJ25), 6N61 (capistruin), and 3CQZ (α-amanitin).

Lasso peptides are characterized by their [1] rotaxane structure, where an isopeptide bond joins the N-terminus of the core peptide to the side chain of a D or E residue, templating a 7–9 aa macrocyclic ring (Cheung-Lee & Link, 2019; Hegemann et al., 2015; Maksimov et al., 2012; Maksimov & Link, 2014). Since the discovery of the first isolated lasso peptide, microcin J25 (MccJ25), lasso peptides have been a hot topic of research due to both their unique rotaxane structure and diverse bioactivities (Salomón & Farías, 1992). Lasso peptides that inhibit RNA polymerases have all been isolated from Gram-negative proteobacteria, including MccJ25 (Salomón & Farías, 1992; Wilson et al., 2003), capistruin (Knappe et al., 2008; Kuznedelov et al., 2011), citrocin (Cheung-Lee, Parry, Cartagena, et al., 2019), ubonodin (Cheung-Lee, Parry, Zong, et al., 2020), acinetodin (Metelev, Arseniev, Bushin, et al., 2017), and klebsidin (Metelev, Arseniev, Bushin, et al., 2017) (Fig. 9A). Braffman et al. recently reported the crystal structures of microcin J25 and capistruin bound to *E. coli* RNA polymerase (Braffman et al., 2019). MccJ25 binds deep within the secondary channel in a way that is expected to interfere with nucleoside triphosphate (NTP) substrate binding (Fig. 9B, C), in agreement with the partial competitive mechanism of inhibition regarding NTPs reported by Mukhopadhyay et al. (2004). The Y9 residue of MccJ25 is found to be strictly essential for RNA polymerase inhibition (Pavlova et al., 2008), and this residue makes the most extensive set of contacts with RNA polymerase. Additionally, the side chain hydroxyl groups of Y9 and Y20 as well as the C-terminus of G21 of MccJ25 form hydrogen bonds with the RNA polymerase, suggesting their importance in the mode of action of MccJ25. It is worth noting that the Y-YG motif, where the first Y is the first residue of the loop, the second Y is the lower lock residue, and the G is at the C-terminus, is conserved in MccJ25, citrocin, and ubonodin (Cheung-Lee, Parry, Cartagena, et al., 2019; Cheung-Lee, Parry, Zong, et al., 2020; Salomón & Farías, 1992). The C-terminal YG motif is also conserved in the case of acinetodin and klebsidin, where the first residue of the loop is T and F, respectively (Metelev, Arseniev, Bushin, et al., 2017). The importance of this motif will be beneficial in discovering novel antimicrobial lasso peptides targeting RNA polymerase through a precursor-centric genome mining approach (Maksimov et al., 2012). In the case of citrocin and ubonodin, abortive transcription initiation assays with *E. coli* RNA polymerase were carried out to confirm both peptides inhibit transcription initiation (Cheung-Lee, Parry, Cartagena, et al., 2019; Cheung-Lee, Parry, Zong, et al., 2020). On the other hand, in the case of acinetodin and klebsidin, single molecule acoustic force spectroscopy experiment was

carried out to measure the speed at which the nucleotides are getting transcribed, and showed both peptides slowed transcription (Metelev, Arseniev, Bushin, et al., 2017). In contrast, capistruin, lacking the Y-YG motif, binds further from the RNA polymerase active site and does not sterically hinder NTP binding (Fig. 9B, D) (Braffman et al., 2019). This observation is also supported by the fact that capistruin inhibition is partially noncompetitive with respect to NTPs (Braffman et al., 2019). It is also worth noting that the uptake of MccJ25 by its susceptible *Escherichia* and *Salmonella* strains is one of the best studied examples of peptide transport in the RiPPs field, which will be discussed further in the Cellular Uptake of RiPPs section (Mathavan et al., 2014; Salomón & Farías, 1993, 1995).

α-amanitin is one of the deadliest amatoxins found in species of the mushroom genus *Amanita*. Structurally, it is a bicyclic octapeptide with C-to-N (head to tail) condensation of the peptide backbone and a cross-bridge between 6-hydroxytryptophan and *S*-oxy cysteine (Fig. 9A) (Hallen et al., 2007; Luo et al., 2012). It is highly specific for RNA polymerase II, causing cytolysis of hepatocytes (Magdalan et al., 2009). Bushnell et al. first solved the cocrystal structure of α-amanitin and RNA polymerase II (Pol II) from *Saccharomyces cerevisiae*, in which α-amanitin interacts with the bridge helix in RNA polymerase II (Fig. 9B, E) (Bushnell et al., 2002). Binding of α-amanitin allows nucleotide entry to the active site and RNA synthesis but prevents the translocation of DNA and RNA needed to enable the next round of synthesis (Bushnell et al., 2002). Brueckner and coworkers, as well as Keplan et al., have subsequently solved two additional cocrystal structures of α-amanitin with *S. cerevisiae* Pol II. Both structures suggested α-amanitin traps the trigger loop (TL) and shifts the bridge helix (Brueckner & Cramer, 2008; Kaplan et al., 2008). The importance of the hydroxyl group of the hydroxyproline residue 2 is highlighted by Brueckner and coworkers, which binds not only to the bridge helix residue E822, but also interacts with the TL residue H1085, and a possible contact with N1082, which binds the bridge helix residue E826 (Brueckner & Cramer, 2008). This observation also agrees with previous analysis on α-amanitin variants, where the hydroxyl group of the hydroxyproline residue 2 has been shown to be crucial for its activity (Wienland & Faulstich, 1991; Zanotti et al., 2009).

Other than targeting the RNA polymerase, linear azo(lin)e peptide (LAP) family member microcin B17 (MccB17) targets DNA gyrase, which can indirectly affect RNA transcription (Booker et al., 2010; Higgins et al., 1988; Peter et al., 2004; Rovinskiy et al., 2012). The mechanism of action of MccB17 will be further detailed in the RiPPs with Other Mechanisms of Action section.

## Translation-Inhibiting RiPPs

Following the production of mRNA, translation is the process during which the ribosome synthesizes proteins by decoding the mRNA. The complementary tRNA anticodon sequences bind to the mRNA codon and the amino acids carried by the individual tRNAs are linked together to form the elongated polypeptides. Diverse classes of RiPPs, including LAPs, bottromycins, thiopeptides, microcin C (McC), have been shown to target various machinery required for this essential process, including the ribosome (Harms et al., 2008; Metelev, Osterman, Ghilarov, et al., 2017; Otaka & Kaji, 1976, 1981, 1983; Travin et al., 2019), the elongation factor Tu (EF-Tu) (Heffron & Jurnak, 2000; Morris et al., 2009), and the aspartyl-tRNA synthetase (AspRS) (Kazakov et al., 2008; Metlitskaya et al., 2006; Piskunova et al., 2017; Ran et al., 2017).

**Fig. 10.**
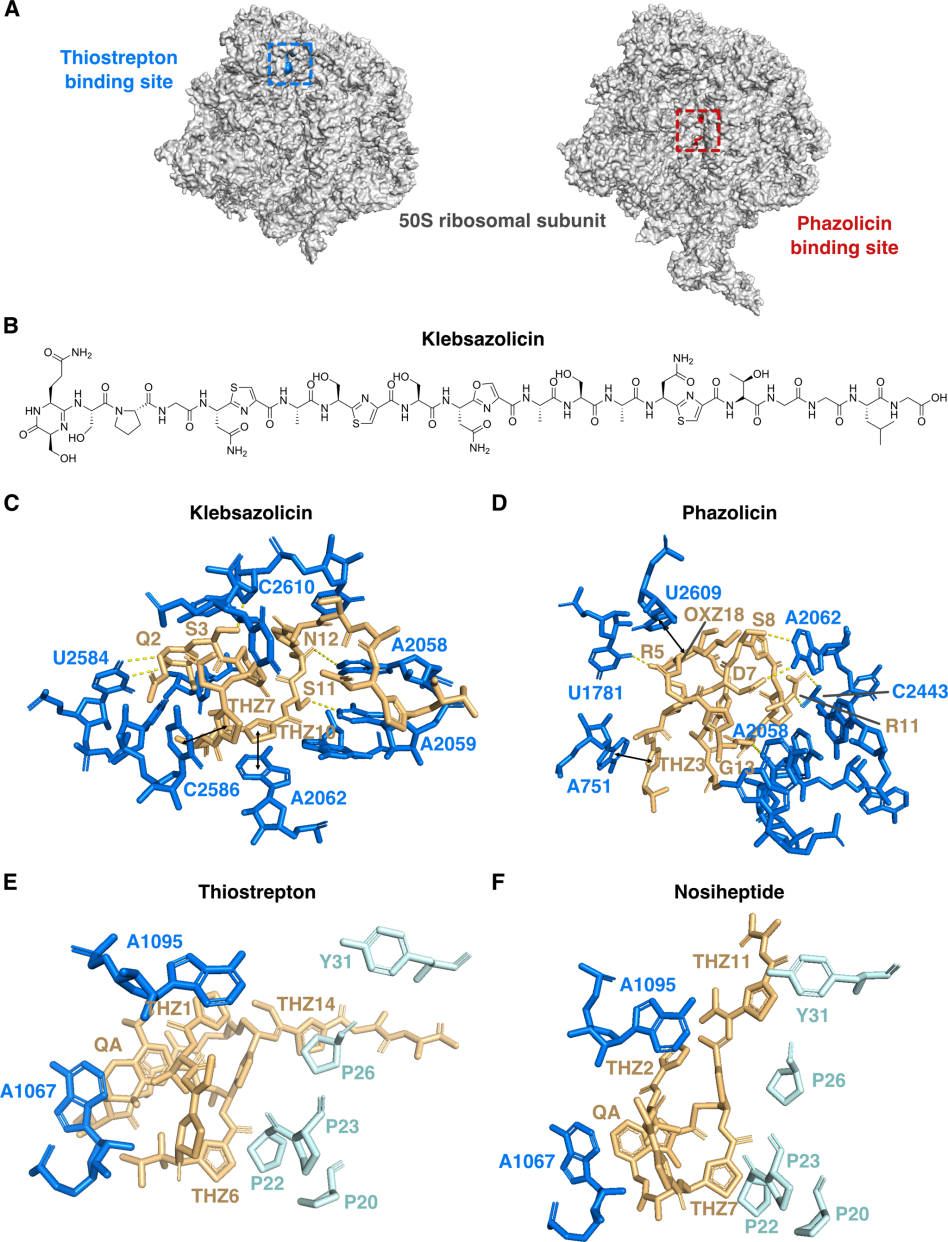
Examples of RiPPs that inhibit translation. (A) Crystal structures of bacterial ribosomes with thiopeptide thiostrepton binding site (PDB file 3CF5) colored in blue and linear azo(lin)e peptide (LAP) phazolicin binding site colored in red (PDB file 6U48). (B) Chemical structure of the LAP klebsazolicin. (C) Key interactions between klebsazolicin (gold) and 23S rRNA (blue), drawn from PDB file 5W4K. Dashed lines are H-bonds (yellow), and double-sided arrows represent π stacking. (D): As in part C, but for the phazolicin:ribosome interaction, drawn from PDB file 6U48. (E) Key interactions between thiostrepton (gold), 23S rRNA (blue), and ribosomal protein L11 (cyan) drawn from PDB file 3CF5. (F) As in part E, but for the nosiheptide:ribosome interaction, drawn from PDB file 2ZJP. THZ: thiazole, OXZ: oxazole, QA: quinaldic acid.

RiPPs that inhibit the ribosome include LAPs, thiopeptides, and bottromycins, including klebsazolicin (Metelev, Osterman, Ghilarov, et al., 2017), phazolicin (Travin et al., 2019), thiostrepton (Bagley et al., 2005; Kelly et al., 2009; Liao et al., 2009), nosiheptide (Shoji et al., 1976) micrococcin (Cundliffe & Thompson, 1981), and bottromycin A2 (Otaka & Kaji, 1976, 1981, 1983). Other than bottromycin A2, crystal structures of these peptides bound to ribosome have been solved (Harms et al., 2008; Metelev, Osterman, & Ghilarov, et al., 2017; Travin et al., 2019). Klebsazolicin and phazolicin belong to LAPs, a family of RiPPs defined by the presence of heterocycles derived from C, S, and T residues in the precursor peptide (Cox et al., 2015). Metelev et al. have crystallized the *Thermus thermophilus* 70S ribosome in the presence of klebsazolicin, mRNA, and A-, P-, and E-site tRNAs, where klebsazolicin is curled to a compacted form in the peptide exit channel on the large ribosomal subunit (Fig. 10B, C) and serves as a compelling example of how flexible RiPPs can be when bound to their molecular targets (Metelev, Osterman, Ghilarov, et al., 2017). Klebsazolicin forms extensive contacts with 23S rRNA of the ribosome; the first and the second thiazole rings (Thz7 and Thz10) stack over C2586-C1782 and A2062-m^2^A2503 base pairs, respectively, and the hydroxyl groups of the S1 and S9 side chain form H-bonds with U2585 and U2506, respectively (Fig. 10C) (Metelev, Osterman, & Ghilarov, et al., 2017). On the other hand, Travin et al. crystallized phazolicin bound to *E. coli* 70S ribosome, after numerous attempts with 70S ribosome from *T. thermophilus*, mRNA, and tRNAs failed (Travin et al., 2019). Similar to klebsazolicin, phazolicin forms a compact globule that obstructs the nascent peptide exit tunnel of the ribosome, and binds further away than klebsazolicin from the peptidyl transferase center of the ribosome (Fig. 10A, D). One striking feature of this cocrystal structure is its complex intramolecular interaction involving the π–π interactions of thiazole12 (Thz12), oxazole21 (Oxz21), Thz6, Oxz18, along with the nucleotide U2609 from the 23S rRNA. Moreover, multiple intermolecular interactions were observed; Thz3, Oxz15, and Oxz18 are involved in π–π stacking with nucleotides A751, C2611, and U2609, respectively, and positively charged side chains of R5 and R11, along with hydrophilic side chains of D7 and S8, form additional H-bonds with the nucleobases and backbone phosphate groups of the 23S RNA.

Thiopeptides are distinguished by their six-membered nitrogen heterocycles, dehydrated amino acids, and thiazole rings formed from dehydrated S/T and cyclized cysteine residues (Bagley et al., 2005; Shen et al., 2019). In contrast to klebsazolicin and phazolicin, thiopeptides that target the ribosome, including thiostrepton, nosiheptide, and micrococcin, target the GTPase-associated center of the ribosome, which is responsible for binding and stimulation of the GTPase activity of translation factors from all phases of translation (Fig. 10A) (Harms et al., 2008; Wilson & Nierhaus, 2005). Harms et al. crystallized the *Deinococcus radiodurans* 50S large ribosomal subunit in complex with thiostrepton, nosiheptide, and micrococcin. Both thiostrepton and nosiheptide were observed in a cleft between the N-terminal domain of ribosomal protein L11 and the tips of H43 and H44 of the 23S rRNA (Fig. 10E, F) (Harms et al., 2008). In particular, Thz6 and Thz14 of thiostrepton stack on P22 and P26 of L11, respectively, and Thz1 forms stacking interactions with A1095 of the 23S rRNA. The binding site of thiostrepton and nosiheptide sterically clashes with the elongation factor-G (EF-G), and both peptides also lock the N-terminus of L11 in a position that will prevent stable interaction between EF-G and the stalk base. In contrast, micrococcin does not bind in the same position as thiostrepton and nosiheptide, with the macrocyclic loop of micrococcin being displaced by ∼6 Å compared to that of thiostrepton. Unlike thiostrepton and nosiheptide, micrococcin was shown to stimulate uncoupled EF-G-dependent GTPase activity (Cameron et al., 2002; Harms et al., 2008; Lentzen et al., 2003). However, due to resolution limitations, the precise molecular interactions of micrococcin with the ribosome were not reported (Harms et al., 2008). Other than inhibiting the ribosome, Bird et al. have recently reported that thiostrepton, nosiheptide, and micrococcin P1, along with thiopeptides thiocillin III, GE37468, and berninamycin, can induce cellular mitophagy (Bird et al., 2020).

Bottromycins are unique peptide antibiotics that contain a macrolactamidine, several nonproteinogenenic amino acids and a thiazole (Gomez-Escribano et al., 2012). Similar to polytheonamides, botthromycins had long been thought to be synthesized through nonribosomal peptide synthetases pathways since their first discovery in 1957 (Waisvisz et al., 1957). In 2012, four studies sequentially confirmed they are indeed RiPPs (Crone et al., 2012; Gomez-Escribano et al., 2012; Hou et al., 2012; Huo et al., 2012). Otaka and coworkers demonstrated that bottromycin A2 targeted the A site of the ribosome by weakening the affinity of peptidyl or aminoacyl-tRNA for the A site (Otaka & Kaji, 1976, 1981, 1983). However, a cocrystal structure of bottromycin with ribosome has yet to be solved.

**Fig. 11.**
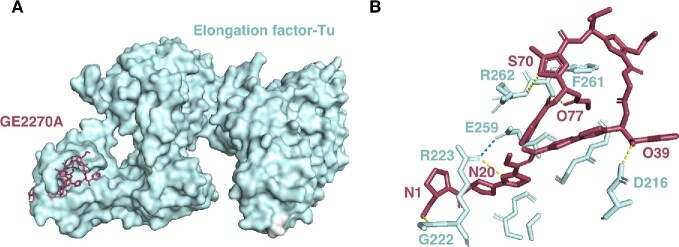
Interaction of thiopeptide GE2270A with elongation factor EF-Tu. (A) Crystal structure of the EF-Tu: GE2270A complex, drawn from PDB file 1D8T. (B) Close up view of the EF-Tu (cyan):GE2270A (magenta) complex. Amino acids from EF-Tu making direct contact with the thiopeptide are labeled. Atoms from GE2270A making direct contact are labeled. Dashed lines are H-bonds (yellow) or a salt bridge (blue) between R223 and E259 that accounts for the high stability of the GE2270A:EF-Tu complex.

The elongation factor thermo unstable (EF-Tu), a guanine nucleotide-binding protein that binds and directs aminoacyl-tRNA to the A-site of the ribosome (Harvey et al., 2019), has also shown to be the molecular target for a group of thiopeptides, including GE2270A (Möhrle et al., 1997; Zuurmond et al., 2000), thiomuracin A (Morris et al., 2009), and amythiamicin A (Shimanaka et al., 1994, 1995). Notably, Heffron and coworkers solved the crystal structure of an EF-Tu complex with GE2270A (Fig. 11A) (Heffron & Jurnak, 2000). In the solved structure, GE2270A binds to a pocket in the second domain of EF-Tu-GDP. The strongest interactions are localized to residues including D216, G222, R223, T256, G257, E259, F261, R262, N273, V274, G275, and L277 (Fig. 11B) (Heffron & Jurnak, 2000). In particular, GE2270A appears to be locked into the binding site by a salt bridge between R223 and E259 of EF-Tu, and five H-bond interactions were observed between GE2270A and EF-Tu (Fig. 11B) (Heffron & Jurnak, 2000).

In addition, the AspRS has been shown to be targeted by McC, a MRTGNAD heptapeptide with an adenosine monophosphate (AMP) analog attached to the *C*-terminus through an *N*-acyl phosphoramidate linkage and *N*-terminal formylation (Severinov & Nair, 2012). Once in the susceptible cells, McC is first deformylated, and then digested to form the toxic processed McC, a nonhydrolyzable analogue of aspartyl-adenylate (Kazakov et al., 2008; Metlitskaya et al., 2006). This mimic inhibits AspRS, which therefore blocks protein synthesis. *In vitro* luciferase gene transcription-translation reaction was carried out to illustrate the mode of action of McC (Metlitskaya et al., 2006). In the presence of processed McC, the exogenously added aminoacylated tRNA^Asp^ was quickly used up, and the second addition of aminoacylated tRNA^Asp^ led to a further increase in luciferase production (Metlitskaya et al., 2006). In the meantime, an orthogonal experiment showed that *E. coli* cells overproducing *D. radiodurans* AspRS were resistant to McC (Metlitskaya et al., 2006). Metlitskaya et al. further illustrated that the processed McC with the aminopropyl group was shown to be approximately 10-fold more potent than the variant without this modification (Metlitskaya et al., 2009).

## RiPPs with Other Mechanisms of Action

Other than the aforementioned mechanisms of action by various RiPPs, there are some notable exceptions that do not fit these categories. The lasso peptide lassomycin is active against multidrug resistant *Mycobacterium tuberculosis* by targeting the Hsp100 family chaperone ClpC1 (Gavrish et al., 2014). ClpC1 complexes with the proteases ClpP1 and ClpP2 to form a protein degradation machine similar to the proteasome. Six resistance mutants of *M. tuberculosis* were raised, and all of them showed mutations in the *clpC1* gene, which encodes the subunit of the hexameric ATPase complex, ClpC1 (Gavrish et al., 2014). In the presence of low concentrations of lassomycin, ATP hydrolysis by ClpC1 increased up to 7- to 10-fold, and this activation by lassomycin showed an apparent *K*_d_ of 0.41 μM, which resembles its MIC value against *M. tuberculosis* (Gavrish et al., 2014). However, lassomycin completely eliminated the ability of ClpC1P1P2 to degrade casein (Gavrish et al., 2014).

Microcin B17 (MccB17) is an *E. coli* antibiotic that inhibits DNA gyrase, an essential bacterial enzyme that catalyzes the ATP-dependent negative supercoiling of double-stranded closed circular DNA (Reece & Maxwell, 1991). Inhibition of DNA gyrase would result in changes in the average supercoil density, which would alter the efficiency and phenotype of proteins involved in transcription (Booker et al., 2010; Higgins et al., 1988; Peter et al., 2004; Rovinskiy et al., 2012), DNA replication (Pang et al., 2005), chromosome segregation (Champion & Higgins, 2007; Ogura et al., 1990), and transposition (Sternglanz et al., 1981). MccB17 binds to the gyrase(A_2_B_2_)–DNA complex in a way that interrupts, but does not completely inhibit the strand-passage reaction, and stabilizes the conformation of the enzyme where the DNA is cleaved and covalently bound to the enzyme (Collin & Maxwell, 2019; Zamble et al., 2001). Although a structure of MccB17 bound to DNA gyrase has yet to be solved, MccB17 has been shown to inhibit DNA supercoiling while still allowing DNA cleavage *in vitro* (Zamble et al., 2001). MccB17 variant studies suggest that the thiazole-oxazole motif at position 39 and oxazole-thiazole motif at position 54 are crucial for its antimicrobial activity (Collin et al., 2013; Collin & Maxwell, 2019).

Sporulation killing factor (SKF) is a 26-residue sactipeptide produced by *Bacillus subtilius* Py79 and *B. subtilis* 168, which is characterized by its thioether bond between C4 and the α-carbon of M12 (Flühe et al., 2013; Gonzalez-Pastor, 2003; Grell et al., 2018). When a *B. subtilis* population is deprived of nutrition, the regulatory protein Spo0A activates the expression of the *skf* and *sdp* operons, which results in the production of SKF and the sporulation delay protein, respectively. Both products are exported, and Spo0A-inactive cells are lysed due to the extracellular SKF (Engelberg-Kulka, 2003; Gonzalez-Pastor, 2003; Liu et al., 2010). This process releases additional nutrients for Spo0A-active *B. subtilis* subpopulation, enabling them to delay the main sporulation event (Flühe et al., 2013; Gonzalez-Pastor, 2003).

Thiopeptide siomycin A targets oncogenic transcription factor Forkhead box M1 (FoxM1), a therapeutic target expressed in a majority of human tumors (Bhat et al., 2009; Laoukili et al., 2007; Radhakrishnan et al., 2006). Siomycin A inhibits expression and transcriptional activity of FoxM1, but not other members of the Forkhead box family. Bhat et al. have shown that siomycin A inhibits the growth and induces potent apoptosis in leukemia CEM, HL60, U937 and liver Hep-3B, Huh7, and SK-Hep cancer cells (Bhat et al., 2009). The apoptosis induced by siomycin A correlates with the suppression of FoxM1 expression, while overexpression of FoxM1 partially protected cancer cells from siomycin-mediated death (Bhat et al., 2009). It is worth noting that thiostrepton, a structurally similar thiopeptide that has shown to inhibit ribosome, also shows similar activity as siomycin A against FoxM1 (Bhat et al., 2009).

## Cellular Uptake of RiPPs

The bioactivity of RiPPs is a product of their ability to first access and then bind their target. We have focused thus far in this review on direct interactions between RiPPs and their cellular targets, but the mechanisms by which the RiPPs are delivered to their targets equally impact bioactivity. Consider the lasso peptides that inhibit RNA polymerase and the thiopeptides that inhibit the ribosome. In both scenarios, the target is cytoplasmic, which means that the exogenous peptides must cross the cytoplasmic membrane and, in the case of a Gram-negative target cell, the outer membrane as well before reaching the intracellular target. Any disruption in this path would curtail bioactivity. In fact, that some RiPPs display a narrow spectrum of activity despite having highly conserved targets has been attributed to whether they can be internalized. However, very little is known about the cellular uptake mechanisms of RiPPs and much of the present knowledge has been confined to the microcin family of RiPPs. Even from these limited studies, a common theme that has emerged is the promiscuous role that endogenous nutrient transporters play in RiPP transport (Mathavan & Beis, 2012).

Genetic screens laid the foundation for uncovering the cellular uptake mechanisms of RiPPs and implicated a small number of proteins as putative membrane transporters. FhuA-mediated uptake of MccJ25 is perhaps the best-understood example of RiPP transport (Mathavan et al., 2014; Salomón & Farías, 1993), but uptake pathways have also been proposed for microcin E492 (Destoumieux-Garzón et al., 2003, 2006; Patzer et al., 2003; Pugsley et al., 1986; Thomas et al., 2004) and microcin B17 (Laviña et al., 1986). As previously discussed, microcin E492 harbors a siderophore-like modification that directs the peptide through TonB-dependent receptors (Destoumieux-Garzón et al., 2003, 2006; Patzer et al., 2003; Pugsley et al., 1986; Thomas et al., 2004). Another factor that is potentially involved in microcin E492 uptake is *semA*, which encodes a protein of unknown function; *semA* mutants were resistant to microcin E492 through an as yet undetermined mechanism (Pugsley et al., 1986). Selection and mapping of microcin B17-resistant mutants similarly revealed that this peptide co-opts a protein native to susceptible cells to cross the outer membrane barrier (Laviña et al., 1986). Based on the genetic evidence, microcin B17 may enter cells through the general porin OmpF, a channel that is normally used for the passive diffusion of small hydrophilic molecules (Laviña et al., 1986). After entering the periplasm, microcin B17 undergoes a second transport step across the inner membrane before delivery to its cytoplasmic target. The polytopic inner membrane protein SbmA has been proposed as the transporter that catalyzes this step (Laviña et al., 1986). The native function of SbmA remains unknown, although SbmA-null mutants are resistant to many antimicrobial peptides including MccJ25 (Salomón & Farías, 1995), which will be discussed shortly, and citrocin (Cheung-Lee, Parry, Cartagena, et al., 2019). Still, it would be counterintuitive for bacteria to evolve a factor that simply serves to sensitize them to harmful agents.

**Fig. 12.**
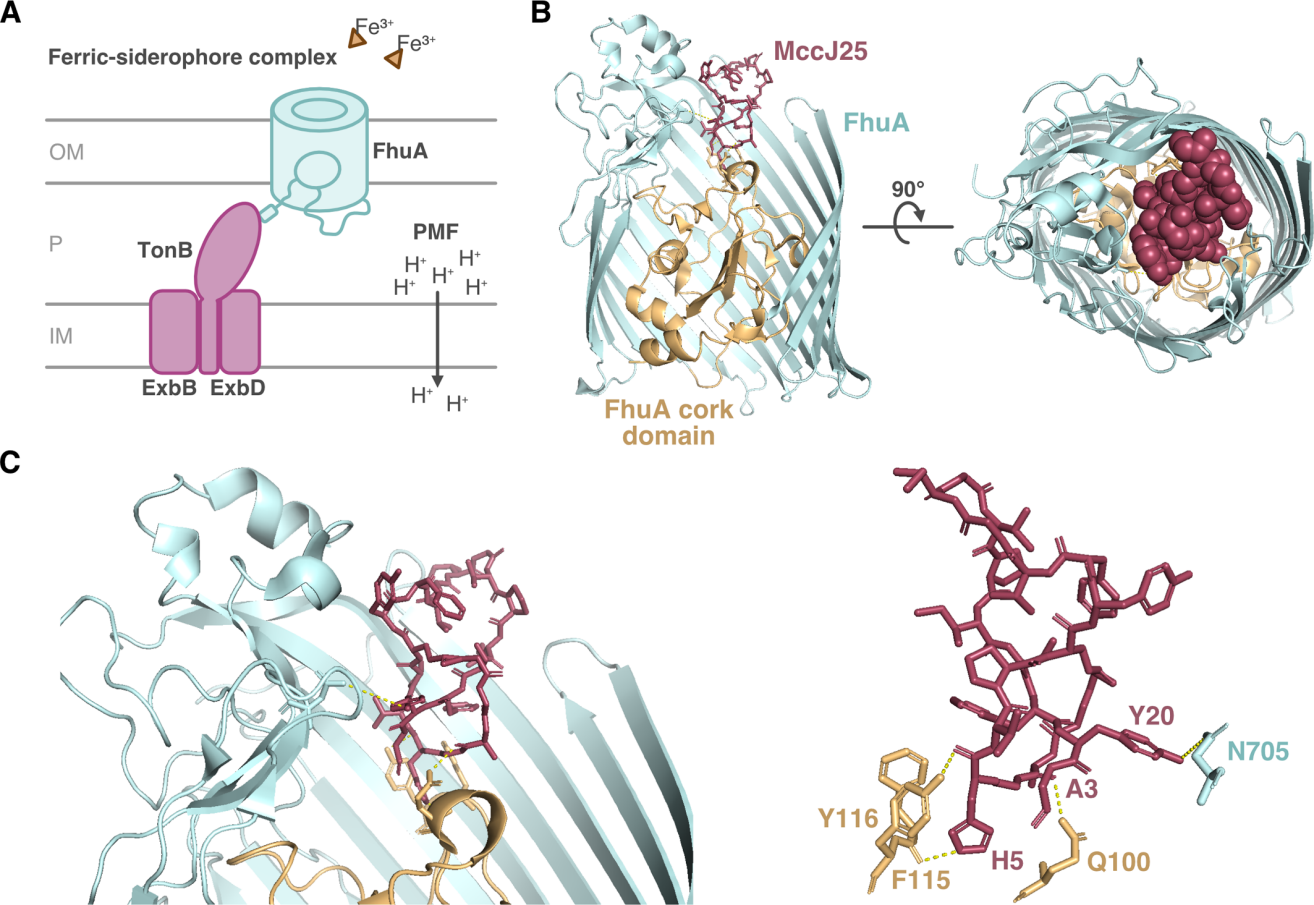
Cellular uptake of lasso peptide microcin J25 (MccJ25) is mediated by the TonB-dependent transporter FhuA. (A) The canonical role of FhuA, an outer membrane (OM) receptor, is transport of iron-siderophore complexes across the OM into the periplasm (P). This energy-dependent process is mediated by the TonB/ExbB/ExbD inner membrane (IM) protein complex and driven by the proton motive force (PMF). (B) Crystal structure of MccJ25 bound to the extracellular face of FhuA. (C) Zoomed-in view of the FhuA:MccJ25 complex showing close contacts between the cork domain (gold) and β-barrel (cyan) of FhuA with MccJ25 (magenta). Dashed lines are H-bonds (yellow). All structure figures drawn from PDB file 4CU4.

Cellular uptake of MccJ25 involves the outer membrane receptor FhuA (Mathavan et al., 2014; Salomón & Farías, 1993) and the inner membrane protein SbmA (Salomón & Farías, 1995). Mutagenesis studies helped to identify the residues that are dispensable for inhibition of RNA polymerase but essential for uptake (Pavlova et al., 2008). The histidine at position 5 within the ring of MccJ25 is important for transport through SbmA (De Cristóbal et al., 2006). The binding of MccJ25 to FhuA is known to involve many more residues, owing to the availability of structural information. FhuA is a TBDT that primarily functions in acquisition of the essential nutrient iron (Braun, 2009; Noinaj et al., 2010). In the environment, iron often exists in the insoluble, oxidized ferric form, that is, iron(III), that bacteria cannot readily internalize. Instead, bacteria produce high-affinity iron-chelating agents called siderophores that bind and carry iron through outer membrane transporters such as FhuA. Transport is coupled to the proton motive force via the TonB-ExbB-ExbD inner membrane complex, which spans the periplasm and directly contacts the transporter (Fig. 12A). FhuA is a monomeric β-barrel protein comprising 22 antiparallel β-strands that wrap around to form a central pore (Ferguson et al., 1998; Locher et al., 1998). The β-strands are connected by short turns on the periplasmic face of the barrel and by long loops on the extracellular face. The central pore is normally gated because the N-terminus of FhuA forms a plug or cork-like domain that inserts from the periplasmic side and occludes the cavity. Binding of a ligand to FhuA displaces the plug and signals TonB to drive transport across the now-continuous channel (Pawelek et al., 2006).

The natural cargo of FhuA is the hydroxamate siderophore ferrichrome, but FhuA also moonlights as a receptor for many foreign agents including bacteriophages and colicin toxins (Braun, 2009). MccJ25 likewise sneaks into susceptible cells through FhuA. MccJ25 docks on the exposed extracellular loops of FhuA but also forms contacts with the β-barrel and cork domain, effectively obstructing the pore near the space that ferrichrome normally occupies (Fig. 12B). This interaction involves the MccJ25 loop region, which is oriented toward the extracellular space (Destoumieux-Garzón et al., 2005; Mathavan et al., 2014; Socias et al., 2009). Upon FhuA binding, the MccJ25 loop is remodeled into a more open configuration relative to its structure in solution (Mathavan et al., 2014). The plane of the MccJ25 8-membered ring lies vertical to the membrane surface and does not undergo major conformational change in the presence of FhuA (Mathavan et al., 2014) The 2.3-Å FhuA-MccJ25 cocrystal structure showed that in addition to abundant hydrophobic contacts across the length of the peptide, three key hydrogen bonds are formed between the MccJ25 ring and the FhuA cork domain (Fig. 12C) (Mathavan et al., 2014). The histidine at position 5 within the MccJ25 ring contributes two of the three hydrogen bonds; it is bonded to the FhuA cork domain residues Y116 and F115 (Mathavan et al., 2014). Moreover, the side chain of this H5 residue is buried in the FhuA pore and is critical for FhuA recognition because an MccJ25^H5A^ mutant has reduced binding affinity (Mathavan et al., 2014). These specific contacts may explain the narrow spectrum activity of MccJ25, as only certain FhuA homologs are capable of MccJ25 uptake (Vincent et al., 2004).

Nature has evolved RiPPs with diverse modes of action. It is remarkable that RiPPs have also evolved to exploit the native receptors of target cells for entry. To repurpose RiPPs into potent antibiotics, one must know the range of bioactivity and a first step toward this goal is to understand cellular uptake. How can we predict what the transporters are in target cells? Early studies on the production and uptake of MccJ25 may provide some clues. Iron deprivation was shown to increase MccJ25 production (Salomón & Fariás, 1994) and MccJ25 uses an iron transporter to enter cells (Mathavan et al., 2014; Salomón & Farías, 1993). This connection may not be a mere coincidence and perhaps suggests that finding the signals which regulate biosynthesis of RiPPs in producer cells can lead us to discovering their transporters in target cells.

## Conclusion

In this review, we have described the molecular mechanisms of action of a variety of different RiPPs for which detailed structural or mechanistic information is available. RiPPs have several defining structural features. First, RiPPs tend to be larger in size than natural products from other classes such as polyketides and nonribosomal peptides. Second, many (though not all) RiPPs exhibit some form of macrocyclization, which results in the RiPP having a more compact structure than a comparable linear peptide. Third, by definition, many RiPPs include chemical moieties not found in the canonical set of amino acid building blocks. All of these features of RiPPs contribute to their mechanisms of activity.

With regards to the size of RiPPs, lasso peptides like capistruin and MccJ25 are sufficiently bulky to occlude most of the secondary channel of RNAP (Fig. 9). The large size of klebsazolicin allows for it to coil up and occupy a large pocket within the 23S rRNA (Fig. 10). Similarly, the large size of polytheonamide B is just the right size for a single molecule of this peptide to span the plasma membrane (Fig. 4). Many RiPPs are too large to passively cross the permeability barriers of bacteria and other cells. While some RiPPs have deduced ways to hijack energy-coupled transport systems (Fig. 12), many other RiPPs simply exert their bioactivity via interactions at the cell envelope instead.

The macrocyclization of RiPPs likely plays a role in generating the right pharmacophore for target engagement. In the lasso peptide MccJ25, the Y9 and Y20 residues make crucial contacts with RNAP (Fig. 9A). These amino acids are in close proximity in the threaded lasso form of MccJ25, but are not near each other in either a linear or unthreaded peptide. Indeed, macrocyclization via the mechanical bond in MccJ25 is critical for the antimicrobial activity of this peptide (Wilson et al., 2003). Head-to-tail cyclization of the bacteriocins (Figs 6 and 7) likely pre-pays some of the entropic penalty associated with membrane binding and pore formation. Another likely role of macrocyclization in RiPPs has more to do with the complex, protease-laden environment into which RiPPs are exported. The organisms that produce RiPPs live in a vast variety of different environments, including dense, complex microbiomes such as soil and the human gut. The macrocyclization of RiPPs allows for enhanced stability of the RiPP relative to a linear peptide in these hostile environments.

Finally, the posttranslational modifications inherent to RiPPs play specific roles in their mechanism of action. The alternating l- and d-amino acids of polytheonamide B give rise to the unusual β-spiral structure that allows it to function as an ion channel (Fig. 4). The thioether rings of nisin help form a cage that binds the pyrophosphate moiety of lipid II (Fig. 3). The aromatic thiazoles and oxazoles in klebsazolicin and phazolicin mediate numerous π–π stacking interactions with ribosomal RNA (Fig. 10).

Structurally diverse RiPPs with novel mechanisms of action will continue to be discovered for decades to come. In the past, it took many years and often the effort of multiple groups to reach an understanding of the mechanism of action following initial isolation of the natural product. As mentioned in the introduction, bioinformatic genome mining has quickened the pace of RiPP discovery. However, these genome mining approaches do not come with a guarantee of bioactivity. Even with bioactivity, determination of the molecular mechanism of action of a RiPP can be arduous work, requiring expertise in genetics, biochemistry, and structural biology. This work is important, however, if RiPPs are to progress to the clinic as antibiotics or cytotoxic payloads. Going forward, we propose that integration of detailed mechanism of action information with genome mining is a way to prioritize the hits from the avalanche of new genome mining data to improve the chances of isolating bioactive RiPPs.
